# Distinct Signal Transduction Pathways Downstream of the (P)RR Revealed by Microarray and ChIP-chip Analyses

**DOI:** 10.1371/journal.pone.0057674

**Published:** 2013-03-04

**Authors:** Daniela Zaade, Jennifer Schmitz, Eileen Benke, Sabrina Klare, Kerstin Seidel, Sebastian Kirsch, Petra Goldin-Lang, Frank S. Zollmann, Thomas Unger, Heiko Funke-Kaiser

**Affiliations:** Center for Cardiovascular Research (CCR)/Institute of Pharmacology, Charité - Universitätsmedizin Berlin, Berlin, Germany; Temple University, United States of America

## Abstract

The (pro)renin receptor ((P)RR) signaling is involved in different pathophysiologies ranging from cardiorenal end-organ damage via diabetic retinopathy to tumorigenesis. We have previously shown that the transcription factor promyelocytic leukemia zinc finger (PLZF) is an adaptor protein of the (P)RR. Furthermore, recent publications suggest that major functions of the (P)RR are mediated ligand-independently by its transmembrane and intracellular part, which acts as an accessory protein of V-ATPases. The transcriptome and recruitmentome downstream of the V-ATPase function and PLZF in the context of the (P)RR are currently unknown. Therefore, we performed a set of microarray and chromatin-immunoprecipitation (ChIP)-chip experiments using siRNA against the (P)RR, stable overexpression of PLZF, the PLZF translocation inhibitor genistein and the specific V-ATPase inhibitor bafilomycin to dissect transcriptional pathways downstream of the (P)RR. We were able to identify distinct and overlapping genetic signatures as well as novel real-time PCR-validated target genes of the different molecular functions of the (P)RR. Moreover, bioinformatic analyses of our data confirm the role of (P)RŔs signal transduction pathways in cardiovascular disease and tumorigenesis.

## Introduction

The (pro)renin receptor (denoted as (P)RR or RER) constitutes a novel component of the renin-angiotensin system (RAS) [1] and has attracted much attention in recent years due to its versatile functions. More than 100 years after the studies by Tigerstedt and Bergman discovering renin [2] a second function was assigned to this enzyme, the binding to the (P)RR and the ability to induce a signal transduction cascade independent from the generation of angiotensin II [1], [3]. This intrinsic activity of renin and also prorenin as ligands at the (P)RR triggers the activation of MAP (mitogen-activated protein) kinases (MAPKs) p42/44 and p38 [4]. We have demonstrated that the transcription factor promyelocytic leukemia zinc finger (PLZF) is a protein-protein interaction partner of the (P)RR, a repressor of the (P)RR promoter and can mediate pro-proliferative/anti-apoptotic cellular effects of renin and prorenin [5], [6]. Senbonmatsu et al. were able to demonstrate that the nuclear translocation of PLZF can be inhibited by the small molecule Genistein [7].

In addition to these non-catalytic (i.e., ligand) effects of (pro)renin, binding of renin to the (P)RR increases its catalytic efficiency whereas binding of prorenin non-proteolytically demasks its enzymatic activity [1]. Furthermore, a soluble isoform of the (P)RR has been described which corresponds to the extracellular (intravesicular) part of this receptor. This isoform is generated by the action of furin and/or ADAM19 [8], [9], [3]. The cleavage product (i.e., the transmembrane and cytoplasmic portion of the (P)RR) most likely corresponds to the vacuolar proton-translocating ATPase (V-ATPase) membrane sector-associated protein M8-9 (ATP6AP2) because the C-terminal 69–100 amino acids of the (P)RR are identical to ATP6AP2; moreover, the (P)RR protein is encoded by the ATP6AP2 gene [3], [10], [11], [12]. The M8-9 domain of the (P)RR is an accessory subunit of the V-ATPase multiprotein complex [13]. M8-9 function is *per se* (pro)renin-independent because (pro)renin-binding is mediated by the extracellular part of the (P)RR [14], [15]. Furthermore, the (P)RR renin-independently exerts a crucial function regarding Wnt signaling which can be inhibited by the specific V-ATPase inhibitor bafilomycin A1 [16]. In addition, wild-type renal podocytes treated with bafilomycin A1 show morphologic and pH changes similar to podocytes with a (P)RR deletion [17]. Consistently, bafilomycin A1 mimics the phenotype (regarding vacuoles accumulation) observed in (P)RR-deficient cardiomyocytes [18]. Finally, mutations in genes encoding V-ATPase subunits cause a similar phenotype in zebrafish as mutagenesis of the (P)RR gene [11]. (Pro)renin-independent (i.e., constitutive) effects of the (P)RR on cell number have also been shown by our group recently (submitted data).

Several publications of independent groups have demonstrated that the (P)RR is mediating cardiac and especially renal as well as ophthalmological end-organ damage independently of angiotensin II [3], [19]. Regarding oncology, the (P)RR ligand prorenin [20], the receptor itself [10], [21] as well as its adaptor protein PLZF [22] and the Wnt receptor frizzled 8 [16] are associated with tumorigenesis.

**Figure 1:**
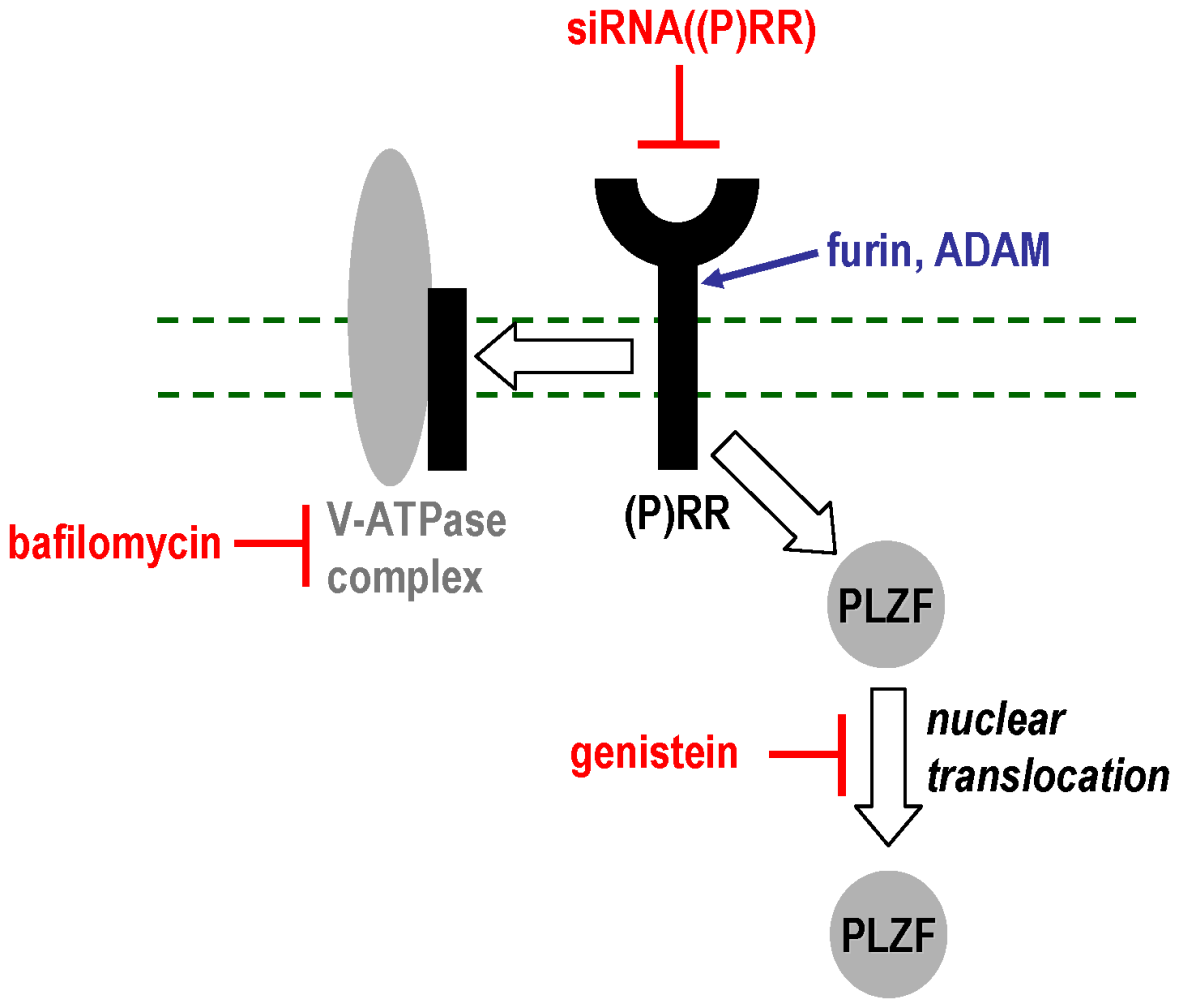
Signal transduction of the (P)RR. Schematic overview of the (P)RR-PLZF signal transduction pathway and the interventions performed (red) in this study. Furin and ADAM are capable of cleaving full-length (P)RR into the soluble (P)RR isoform and a V-ATPase-associated identity.

As discussed below, expression profiling of the transcriptome downstream of the (P)RR has already been performed but only concerning ligand-mediated effects [23], [24]. Therefore, the objectives of this study were to dissect the (P)RR signal transduction cascade according to the downstream mRNA targets and also downstream protein-DNA interactions of its distinct components (PLZF, V-ATPase-associated isoform *versus* non-V-ATPase function of the (P)RR; Figure 1) by using a microarray- and ChIP-chip-based approach. This would give insights into putative modular functions of the (P)RR pathway. Furthermore, we aimed to identify single transcripts exhibiting high dynamic windows (i.e., signal-to-background ratios) to be used as future robust biomarkers.

## Materials and Methods

### Cell Culture

KELLY wild type cells (DSMZ, Braunschweig, Germany) were grown in RPMI 1640 medium (Life Technologies, Darmstadt, Germany). HEK293T (American Type Culture Collection (ATCC), Manassas, VA, USA) cells were cultivated in DMEM high glucose (Life Technologies). All media contained 10% fetal bovine serum (Life Technologies), 100 U/ml penicillin and 100 µg/ml streptomycin (A2212, Biochrom, Berlin, Germany). All cell lines were cultivated in a humidified incubator at 5% CO_2_ and 37°C. For microarray experiments, KELLY cells cultured in RPMI1640 (Life Technologies) without starving were incubated with a final concentration of 10 µM genistein (Carl Roth, Karlsruhe, Germany) for 48 h or 1 nM of bafilomycin A1 (Enzo Life Science, Lörrach, Germany) for 48 h. Genistein and bafilomycin A1 were solved in 1% DMSO (final concentration within the medium); 1% DMSO (final) was used as a negative control.

### Generation of Stable Cell Lines

KELLY cells overexpressing PLZF fused to a C-terminal HA-tag were generated as described previously [25]. PLZF overexpression without tag in HEK293T cells was performed with pCEP4 vector (Life Technologies) and the following primers: 5′-gctaggctagcatggatctgacaaaaatgggcatgatccagc-3′ (sense), 5′-cctggatcctcacacatagcacaggtagaggtacg-3′ (antisense). After transfection, HEK293T cells were selected using hygromycin B (250 µg/ml final concentration within the medium; PAA, Pasching, Austria).

### SiRNA Experiments

SiRNA experiments were performed with siRNA against the (P)RR [5′-gcuccguaaucgccuguuu-3′ (sense strand)] or scrambled control siRNA [5′-uuuaccgucgccuugagcu-3′ (sense strand)] (Eurogentec, Köln, Germany) using Hiperfect (Qiagen, Hilden, Germany) and a final siRNA concentration of 25 nM. SiRNA was transfected twice (48 h and 24 h before harvest). Knockdown of (P)RR by siRNA was confirmed by real-time PCR and Western blotting.

### RNA Isolation and Microarray Hybridization

Total RNA was extracted using the Nucleospin-RNA-II (Macherey-Nagel) according to the manufacturer’s instructions. In addition, samples were treated with DNAse I (Promega, Mannheim, Germany). The concentration of the RNA was quantified spectrophotometrically (Nanodrop, ND 1000, Peqlab, Erlangen, Germany) and the RNA integrity was analysed using the Agilent RNA 6000 Nano Kit and the Bioanalyzer 2100 (Agilent, Santa Clara, USA).

Transcriptome analyses were carried out using Genechip Human Gene 1.0 ST Arrays or Genechip Human Exon 1.0 ST Arrays (only in the case of siRNA and PLZF-HA overexpressing experiments) (both Affymetrix, Santa Clara, USA) and the Genechip Whole Transcript (WT) Sense Target Labeling Assay (Affymetrix) according to the manufacturer’s manual. In detail, ribosomal RNA (rRNA) was reduced from 1 µg total RNA using the Ribominus Transcriptome Isolation Kit (Human/Mouse; Life Technologies) to minimize the background and to increase the array sensitivity and specificity. From the reduced RNA double-stranded cDNA was synthesized with random hexamer primer coupled with a T7 promoter sequence using Superscript II and DNA polymerase I. The cDNA was used as a template for *in vitro* transcription amplification with T7 RNA polymerase obtaining cRNA that was reverse complementary to original mRNA. For second cycle cDNA synthesis, random primers were used in reverse transcription to convert the cRNA into single-stranded DNA in sense orientation using RNA polymerase II (pol II). During the second cycle first-strand reverse transcription reaction, dUTP was incorporated into the cDNA. Subsequently, the single-stranded DNA sample was fragmented by treatment with a combination of uracil DNA glycosylase (UDG) and apurinic/apyrimidinic endonuclease 1 (APE 1). Fragmented DNA was labeled with terminal deoxynucleotidyl transferase (TdT) reaction with a labeling reagent covalently linked to biotin and hybridized to the array. Hybridization, washing, and scanning were carried out using the Hybridization Kit and the Wash and Stain Kit (both Affymetrix).

Each chip was hybridized with the cDNA derived from the mRNA of a single cell culture flask or from independent pools of 3 independent mRNA extractions in the case of genistein (n = 6 independent RNA isolations for 2 microarrays) and bafilomycin (n = 9 independent RNA isolations for 2 microarrays) incubations.

### Statistical Analysis of Microarray Experiments

The quality control and primary data analysis were performed with Expression Console software (Affymetrix). The Chipinspector software (release 2.1; Genomatix, Munich, Germany) and ElDorado (08-2011; Genomatix) served for further data analyses and probe annotation. Raw data were normalised on the single probe level and subsequent pairwise comparison analyses of the expression values from the experimental condition versus control conditions were performed based on a single-sided permutational t-test analysis [26]. Fold change is defined as the signal of the experimental condition divided by the signal of control condition (i.e., without logarithmic calculus). Fold change thresholds were set to values of ≥1.5 or ≤0.67. Experimental conditions were e.g. PLZF overexpression or compound incubations; control conditions were e.g. DMSO or transfection of insertless vectors. A fold change of 2 means that the respective mRNA level was doubled by the intervention; 0.5 indicates halving. Depending on the signal intensities of the individual microarrays, false discovery rate (FDR) was set to 0.5% for Ma(bafi), 5% for Ma(geni), 2% for Ma(si(P)RR), 1% for Ma(PLZF, H), 10% for positive and 23.4% for negative data points of Ma(PLZF; K). The fold changes of the two latter were set to ≥2 or ≤0.5. The minimal probe coverage (the number of significant single probes that detect a transcript) was set to 3.

The microarray data are available in the GEO database (www.ncbi.nlm.nih.gov/geo/) under the accession numbers GSE39961 to GSE39965.

### Chromatin-immunoprecipitation (ChIP)

KELLY and HEK293T cells were fixed at a conﬂuence of about 90% using 1% formaldehyde (Merck, Darmstadt, Germany) in PBS (Life Technologies) for 7 min at 37°C. Subsequently, cells were rinsed twice with ice-cold 1×PBS, scraped off in 1×PBS and centrifuged for 5 min at 660 g. Each pellet was resuspended in 2.5 ml lysis buffer (1% SDS, 50 mM Tris–HCl, 1X complete protease inhibitor cocktail (Roche, Mannheim, Germany), 5 mM EDTA (final concentration); pH 8.1), followed by a 20 min incubation on ice. Sonification, immunoprecipitation and reversal of crosslink were performed according to Bryant and Ptashne [27] using 3 µg of the following antibodies: anti-PLZF (mouse mAb (2A9), Calbiochem/EMD Millipore, Darmstadt, Germany), anti-RNA polymerase II (raised against a peptide mapping at the N-terminus; sc-899 X, Santa Cruz Biotechnology, Santa Cruz, USA) and anti-IgG (rabbit, 2729S, Danvers USA). Sonification was carried out using the Sonoplus HD 2070/UW 2070 sonifier with the tip MS 72 (Bandelin Electronic, Berlin, Germany) and a constant duty cycle with output control (power %) of 100 for 20 s (twice) and output control of 30% for 20 s (three times). After reversal of crosslink, DNA was purified using QIAquick PCR Purification Kit (Qiagen, Hilden, Germany). As additional control before ChIP-chip hybridisation, quantitative genomic PCR was performed in technical triplicates using anti-RNA polymerase II precipitates, Go-Taq qPCR Master Mix (Promega) and the following primers located around the transcriptional start site: Human beta-actin promoter: 5′-aatgctgcactgtgcggcga-3′ (sense), 5′-ggcggatcggcaaaggcga-3′ (antisense). Input (total) DNA served as positive control; probes without crosslinking and/or precipitates using the anti-IgG antibody served as negative control(s).

### ChIP-chip Analysis

Prior to hybridization to promoter arrays, efficient chromatin-immunoprecipitations were confirmed by genomic PCR as described above using RNA polymerase II immunoprecipitations and IgG samples. Purified, immunoprecipitated and total DNA were amplified using the Genomeplex Complete WGA Kit (WGA2) (Sigma-Aldrich, St. Louis, USA) and the protocol of O’Geen et al. [28]. The amplified DNA was labeled with Cy3 or Cy5 and hybridized on HD2 Promoter Tiling arrays (human, C7291-00-02, hg18 Deluxe Promoter HX1, Roche Nimblegen, Madison, USA). The regions covered by this promoter chip span from 7250 bp upstream to 3250 bp downstream of the transcriptional start sites (TSS). Analysis and peak identification were performed using Nimblescan software (Roche Nimblegen). The probe sequences were remapped to the human genome (hg19) (Genome Analyzer, Genomatix). Based on this mapping, the clustering of the unique probes resulted in 34,162 regions of which 69% overlapped with promoters. The peak files (general feature format (gff)) of each ChIP-chip experiment were re-clustered using the Next Generation Sequencing (NGS) Analyzer, which uses a sliding window approach with at least three probes in 500 bp, and the Regionminer (release 4.4, homo sapiens, NCBI build 37) software (both Genomatix). Resulting cluster were annotated for their next neighbouring genes 10 kb upstream and downstream of the enriched regions.

The ChIP-chip data are available in the GEO database (www.ncbi.nlm.nih.gov/geo/) under the accession number GSE39960.

### Quantitative (Real-time) PCR Analysis and Western Blotting

RNA was reverse transcribed using M-MLV reverse transcriptase (RNase H minus) and random hexamer primers (both Promega, Mannheim, Germany) according to the manufacturer’s instructions. PCR was performed using GoTaq-Mix (Promega) and primer pairs given in Table S1 in File S1. A reaction without addition of reverse transcriptase (RT-) served as negative control. Data analyses were performed according to the ddCT method. Only real-time PCR runs with a standard deviation below 40% were considered technically valid and were included in the final data analysis given in the results section.

Western blotting was performed as published previously by our group [25].

### Bioinformatic Network Analysis

To reveal functional connections between the regulated transcripts, a network and pathway analysis of identified genes was performed using Ingenuity Pathway Analysis (IPA, version 9.0, release date: 2011-12-14, Ingenuity Systems, Redwood City, USA). Differentially expressed genes (under- and overrepresented transcripts) were classified according to toxicity phenotypes, clinical pathology endpoints and molecular functions using IPA-Tox Analysis and Core Analysis. Results (all categories and functions) were filtered using a p-value cutoff of 1% and a minimum number of involved molecules of 10. The z-score of the predicted activation state calculated by IPA for all analyses was between −2 and +2 indicating that a prediction whether a certain associated disease or process will increase or decrease based on the directions of the transcriptional changes can not be made, except decreased (z-score = −2.3) likelihood for tumorigenesis (Ma(PLZF; H, K)) and increased one (z-score = 2.6) for diabetes (Ma(si(P)RR)).

## Results

### Transcriptome Downstream of the (P)RR

**Figure 2:**
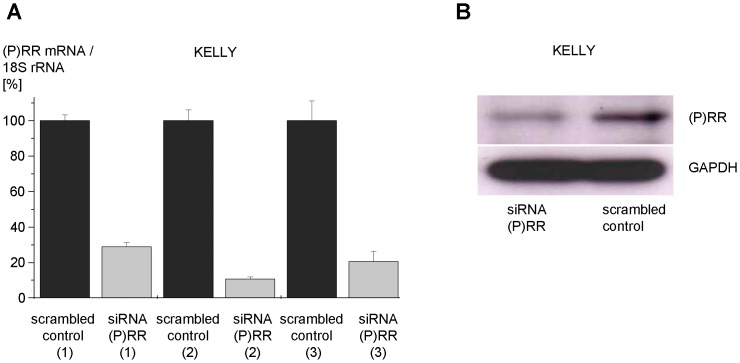
Confirmation of siRNA efficacy in KELLY cells. (A) KELLY cells were transfected with siRNA against (P)RR or scrambled control siRNA. Transcript levels were quantified by real-time PCR in technical triplicates. The numbers 1 to 3 indicate total RNA preparations used for microarray analyses. (B) Total protein was extracted from KELLY cells as used in (A) and subjected to Western blotting using anti-(P)RR (HPA003156, Sigma-Aldrich) and anti-GAPDH antibodies (MAB374, Millipore).

To modularly dissect the transcriptome of the (P)RR-PLZF pathway, we performed a series of microarray experiments summarized in Table 1. Initially, we repressed (P)RR expression by siRNA to identify its transcriptome. This intervention induced changes in mRNA expression of 1652 genes. 1519 of these genes exhibited an increase and 133 genes a decrease in mRNA levels relative to the scrambled control siRNA. The latter includes the renin receptor (ATP6AP2) itself with a fold change of 0.33 (i.e., a repression to 33% on mRNA level; Table S2 in File S1) which is consistent with our control experiments using real-time PCR (repression of the (P)RR to about 10% to 30%, Figure 2A) and Western blotting (repression of the (P)RR to about 40%, Figure 2B). The genes with the highest fold changes under the siRNA((P)RR) intervention as well as target genes previously known from the literature are given in Table S2 in File S1.

**Table 1:** Overview of microarray experiments.

abbreviation	cell type	experimental versus control condition	number ofmicroarrays	differentially transcribed genes
Ma(PLZF; H)	HEK293T	stable overexpression of unttagged PLZF versusstable expression of insertless vector	3	1632
Ma(PLZF; K)	KELLY	PLZF-HA-expression versus insertless vector	2	5073
Ma(si(P)RR)	KELLY	siRNA against (P)RR versus scrambled siRNA control	3	1652
Ma(geni)	KELLY	10 µM genistein versus DMSO	2	636
Ma(bafi)	KELLY	1 nM bafilomycin A1 versus DMSO	3	1788

Bioinformatic network analysis using IPA revealed that the genes downstream of the (P)RR were significantly associated with disease processes such as tumorigenesis (25% of all identified genes), diabetes mellitus (23%) and tissue development (17%) (Table 2).

**Table 2:** IPA analyses of microarray data from experiment Ma(si(P)RR), Ma(bafi), Ma(PLZF; H, K), Ma(geni).

	Ma(si(P)RR)			Ma(bafi)			Ma(PLZF; H, K)			Ma(geni)		
	input:		1652	input:		1788	input:		541	input:		636
function annotation	p-value	n	[%]	p-value	n	[%]	p-value	n	[%]	p-value	n	[%]
tumorigenesis	7.8E–07	420	25	9.9E−03	397	22	6.7E−05	135	25	–	–	–
* gastrointestinal tract cancer*	6.2E−05	122	7	1.7E−04	124	7	2.6E−04	44	8	–	–	–
* colorectal cancer*	6.8E−05	110	7	3.4E−03	104	6	7.2E−05	42	8	–	–	–
* breast cancer*	9.0E−03	101	6	–	–	–	–	–	–	–	–	–
* head and neck cancer*	3.3E−05	81	5	–	–	–	–	–	–	–	–	–
* prostate cancer*	1.6E−03	72	4	–	–	–	2.2E−04	30	6	–	–	–
* brain cancer*	5.3E−06	46	3	–	–	–	–	–	–	–	–	–
* melanoma*	2.2E−03	42	3	–	–	–	5.5E−03	16	3	–	–	–
* non-small cell lung cancer*	2.3E−03	40	2	–	–	–	–	–	–	–	–	–
* glioma*	5.5E−05	31	2	–	–	–	–	–	–	–	–	–
* biliary tract cancer*	9.0E−03	16	1	–	–	–	–	–	–	–	–	–
* non-Hodgkin’s disease*	–	–	–	6.3E−04	43	2	–	–	–	–	–	–
* lymphoid cancer*	–	–	–	6.1E−03	53	3	–	–	–	–	–	–
* chronic B-cell leukemia*	–	–	–	9.6E−03	19	1	–	–	–	–	–	–
* diffuse small-cell lymphoma*	–	–	–	3.3E−03	10	1	–	–	–	–	–	–
* endometrial cancer*	–	–	–	–	–	–	2.2E−04	14	3	–	–	–
diabetes mellitus	2.7E−35	381	23	4.8E−04	264	15	5.5E−07	99	18	6.7E−03	93	15
tissue development	1.0E−07	275	17	4.7E−03	250	14	1.4E−14	120	22	1.2E−03	96	15
* development of connective tissue*	3.8E−04	81	5	–	–	–	–	–	–	3.6E−03	32	5
* vasculogenesis*	2.2E−06	77	5	–	–	–	1.3E−06	33	6	2.1E−03	28	4
* development of bone*	1.7E−04	74	4	–	–	–	4.7E−03	25	5	–	–	–
* cardiogenesis*	1.9E−03	38	2	–	–	–	3.7E−03	15	3	–	–	–
* kidney development*	1.3E−04	31	2	–	–	–	–	–	–	–	–	–
* skin development*	–	–	–	–	–	–	4.2E−03	13	2	–	–	–
* development of brain*	5.3E−04	49	3	–	–	–	1.0E−06	26	5	–	–	–
* development of forebrain*	–	–	–	–	–	–	3.9E−04	12	2	–	–	–
atherosclerosis	1.6E−30	238	14	2.7E−04	154	9	9.3E−08	65	12	5.6E−04	60	9
* coronary artery disease*	1.9E−26	212	13	1.1E−04	143	8	1.3E−07	60	11	1.8E−03	53	8
rheumatoid arthritis	1.2E−13	222	13	–	–	–	4.2E−05	66	12	4.9E−06	79	12
inflammatory bowel disease	8.8E−23	205	12	9.2E−04	139	8	5.1E−06	56	10	4.1E−03	52	8
mood disorder	3.0E−13	178	11	7.2E−04	141	8	3.2E−07	60	11	3.1E−03	53	8
* bipolar disorder*	1.1E−14	166	10	1.9E−03	123	7	2.7E−07	55	10	1.6E−03	49	8
hypertension	4.7E−15	165	10	7.0E−03	117	7	8.0E−05	47	9	–	–	–
Alzheimer’s disease	1.2E−06	122	7	–	–	–	1.9E−05	45	8	2.8E−03	43	7
HIV infection	6.2E−04	115	7	–	–	–	–	–	–	–	–	–
Parkinson’s disease	4.4E−05	82	5	–	–	–	7.2E−03	26	5	–	–	–
amyotrophic lateral sclerosis	2.2E−05	79	5	–	–	–	1.0E−05	33	6	–	–	–
schizophrenia	1.6E−03	69	4	–	–	–	1.0E−03	27	5	–	–	–
endometriosis	3.2E−04	58	4	–	–	–	2.8E−03	21	4	–	–	–
activation of MAP kinase cascade	8.7E−05	27	2	–	–	–	–	–	–	–	–	–
dilated cardiomyopathy	–	–	–	3.5E−04	18	1	–	–	–	–	–	–
hematopoiesis	–	–	–	–	–	–	–	–	–	3.1E−05	43	7
multiple sclerosis	–	–	–	–	–	–	3.2E−03	13	2	–	–	–
psoriasis	–	–	–	–	–	–	5.8E−03	26	5	–	–	–

The significance levels, the number of involved molecules (n) as well as their percentages relative to the input datasets (under- and overrepresented transcripts) are given. The functional classification of a specific gene can be redundant due to the assignment of one gene to more than one category.

### Transcriptional Signature of Bafilomycin

Besides siRNA silencing of the (P)RR, we focused on the V-ATPase-mediated function of this receptor by using the specific V-ATPase inhibitor bafilomycin A1 within our microarray experiments (Ma(bafi) in Table 1).

In total 1788 genes showed an altered mRNA expression, 1364 genes with an increase and 424 genes with a decrease in mRNA levels compared to the DMSO control. The genes with the highest fold changes are given in Table S3 in File S1.

The respective network analysis of the altered transcriptional profile induced by bafilomycin is shown in Table 2. The (patho)physiological functions classified by IPA were tumorigenesis (22% of all genes with an altered mRNA level), diabetes mellitus (15%) and tissue development (14%).

### Comparisons of (P)RR and Bafilomycin Gene Signatures

**Figure 3:**
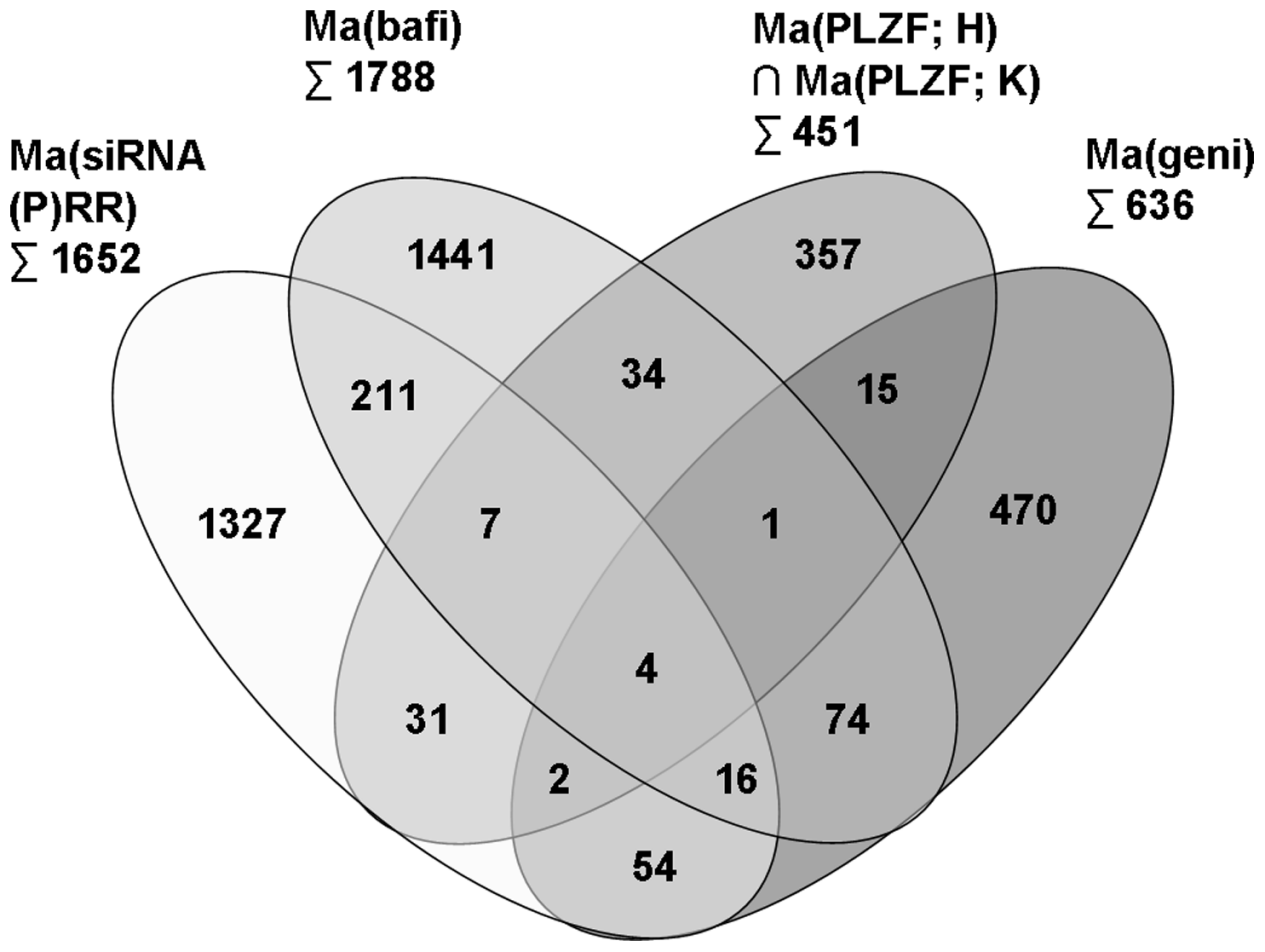
Overlap of intervention-specific transcriptional signatures. Venn diagram displaying the intersections between genes identified as being differentially regulated by microarrays Ma(si(P)RR), Ma(bafi), Ma(PLZF; H) ∩ Ma(PLZF; K) and Ma(geni). Under- and overrepresented transcripts were used as input.

In order to identify M8-9-dependent genes within the bafilomycin-altered transcriptome, we generated an intersection of Ma((P)RR) and Ma(bafi) results which comprised 238 genes (Table 3, Figure 3). These genes are associated with tumorigenesis (28% of all genes with a changed mRNA level), diabetes mellitus (24%) and tissue development (19%) (Table S4 in File S1). Transcripts with the highest fold changes are given in Table S5 in File S1.

**Table 3:** Pairwise intersections of the filtered datasets of the performed microarrays Ma(si(P)RR), Ma(bafi), Ma(PLZF; H) ∩ Ma(PLZF; K) and Ma(geni).

		Ma(si(P)RR)		Ma(bafi)		Ma(PLZF; H) ∩Ma(PLZF; K)		Ma(geni)	
	genes with								
	increased mRNA levels	1519		1364		447		609	
	decreased mRNA levels	133		424		4		27	
	∑ diff. expr. mRNA	1652		1788		451		636	
	**mRNA levels**	**n**	**%**	**n**	**%**	**n**	**%**	**n**	**%**
Ma(siRNA	both increased			124		38		61	
(P)RR)	both decreased			2		0		0	
	increased/decreased			112		6		15	
	**∑ diff. expr. mRNA**			**238**	**13**	**44**	**10**	**76**	**12**
Ma(bafi)	both increased					36		48	
	both decreased					0		6	
	increased/decreased					10		42	
	**∑ diff. expr. mRNA**					**46**	**10**	**95**	**15**
Ma(PLZF; H)	both increased							20	
∩	both decreased							0	
Ma(PLZF; K)	increased/decreased							2	
	**∑ diff. expr. mRNA**							**22**	**5**

Genes with a significantly changed mRNA level based on the defined FDR and fold change values are given. The numbers of concordantly (both increased, both decreased) and inversely (increased/decreased) regulated transcripts are specified. Diff. expr. mRNA: total number of differentially expressed mRNAs.

### Transcriptome of the PLZF

**Figure 4:**
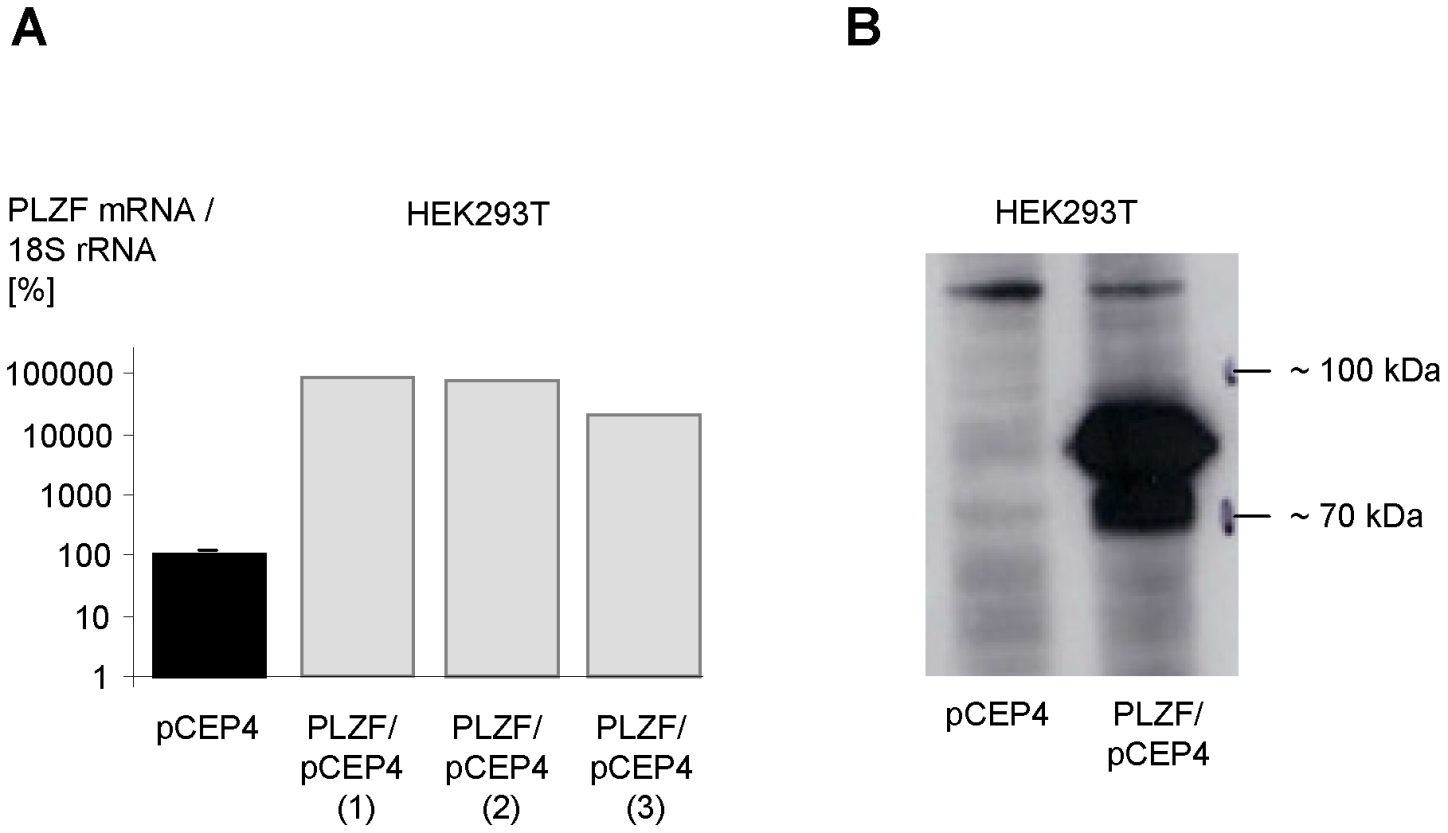
Confirmation of stable PLZF overexpression in HEK293T cells. (A) HEK293T cells were stably transfected with an expression vector encoding PLZF (PLZF/pCEP4) or an insertless control plasmid (pCEP4). Transcript levels were quantified by real-time PCR in technical triplicates. The numbers 1 to 3 indicate total RNA preparations used for microarray analyses. Standard deviations are given with respect to all columns. (B) Total protein was extracted from HEK293T cells as used in (A) and subjected to Western blotting using an anti-PLZF antibody (sc-28319, Santa Cruz Biotechnology).

To investigate genome-wide transcriptional profiles downstream of PLZF in order to analyse the contribution of this transcription factor to transcriptome downstream of the (P)RR (Figure 1), we stably overexpressed PLZF in HEK293T cells followed by microarray expression analysis (Table 1). Overexpression of PLZF was confirmed by quantitative (real-time) PCR and Western blotting (Figures 4A and 4B). From 28,869 human genes analysed, significant changes in the mRNA level were detected for 1632 genes; 1278 showed an increase and 354 a decrease in mRNA levels compared to the cells expressing the insertless vector control. Among the 1278 genes, PLZF (ZBTB16) was detected with a fold change of 21.11 indicating a 21-fold overexpression. Furthermore, the gene encoding for the inhibitor of DNA binding 3 (ID3), which has been mentioned in literature as PLZF-regulated [29], showed a significantly altered mRNA level (Table S6 in File S1). The genes with the highest fold changes are shown in Table S6 in File S1.

We have previously shown that overexpression of HA-tagged PLZF in KELLY cells caused a neuroprotective effect [25]. Therefore, we performed an expression profiling in these cells in order to gain more insight into the transcriptional changes underlying this cellular effect.

In total 5073 genes were detected with altered mRNA levels compared to the control (KELLY cells stably transfected with the insertless vector). 260 of these genes had a decrease and 4813 an increase in mRNA levels. The genes with the highest fold changes in mRNA level are listed in Table S7 in File S1. PLZF (ZBTB16) itself showed a fold change of 56.49. In addition, three genes encoding for the inhibitor of DNA binding 1, 2 and 3 (ID1, ID2 and ID3) exhibited a significantly altered mRNA level (Table S7 in File S1) and have been described as PLZF target genes previously [29]. Furthermore, most mRNAs encoding for the genes of the V-ATPase cassette were found to be underrepresented (fold change = 0.64 (ATP6AP1), 0.63 (ATP6AP2), 0.60 (ATP6V0A2), 0.57 (ATP6V0A4), 0.64 (ATP6V0B), 0.65 (ATP6V1A), 0.66 (ATP6V1B2), 0.59 (ATP6V1C1), 0.61 (ATP6V1C2), 0.62 (ATP6V1D), 0.57 (ATPV1E1), 0.62 (ATP6V1G1), 0.61 (ATP6V1G2), 0,63 (ATP6V1G2-DDX39B), 0.60 (ATP6V1H)).

To estimate the genes regulated by PLZF independently of the cell type and the presence or absence of a tag, an intersection of Ma(PLZF; H) and Ma(PLZF; K) results – denoted as Ma(PLZF; H; K) – was generated comprising 451 genes. The IPA analyses of this dataset revealed a significant involvement of genes associated with tumorigenesis (25%), tissue development (22%), atherosclerosis (12%), among others (Table 2).

### Transcriptional Signature of Genistein

Genistein has been described as an inhibitor of nuclear translocation of PLZF [7] (Figure 1). Gene expression profiles of KELLY cells incubated with genistein (Table 1) revealed 636 genes with an altered mRNA level compared to a DMSO control. 609 genes of these exhibited an increase and 27 a decrease in mRNA levels. The genes with the highest fold changes are given in (Table S8 in File S1).

The respective network analysis of the altered transcriptional profile mediated by genistein is shown in Table 2. The (patho)physiological functions classified by IPA were diabetes mellitus (15%), tissue development (15%) and rheumatoid arthritis (12%).

### Comparisons of Genetic Signatures Downstream of PLZF and Genistein

Since genistein mediates multiple pharmacodynamic effects beside PLZF translocation inhibition and since overexpression might cause unspecific transcriptional effects, we intersected Ma(PLZF; H, K) and Ma(geni) results to validate PLZF target genes (Table 3 and Figure 3). The intersection of Ma(PLZF; H, K) and Ma(geni) results revealed 22 genes (Table 3), of which 20 genes exhibited a concordant regulation (Table S9 in File S1). Two of the 22 transcripts ITGB8 (integrin, beta 8) and SLC4A4 (solute carrier family 4 sodium bicarbonate cotransporter, member 4) with fold changes of 0.57 and 0.53, respectively, exhibited inverse regulation patterns (i.e., upregulation versus downregulation) consistent with the fact that PLZF overexpression and genistein as an inhibitor of PLZF nuclear translocation overtly act via opposite mechanisms.

### Comparisons of Genetic Signatures Downstream of (P)RR and PLZF

The intersection between the microarrays Ma(si(P)RR) and Ma(PLZF; H, K) comprised 44 genes (Table 3, Figure 3). The genes with the highest fold changes are shown in Table S10 in File S1.

The respective network analysis of the intersectional transcriptional profile revealed e.g. tissue development (43%) and diabetes mellitus (36%) as (patho)physiological functions (Table 4).

**Table 4:** Overview of ChIP-chip (Cc) experiments.

experiment	cell type/intervention 1	antibody for IP usedin intervention 1	cell type/intervention 2	antibody for IP used in intervention 2
Cc1	KELLY/stable PLZF overexpression(PLZF-C-HA/pCEP4)	RNA polymerase II	KELLY/stable PLZF overexpression(PLZF-C-HA/pCEP4)	IgG
Cc2	KELLY/stable PLZF overexpression(PLZF-C-HA/pCEP4)	RNA polymerase II	KELLY/stable transfection of insertless vector (pCEP4)	RNA polymerase II
Cc3	KELLY/stable transfection ofinsertless vector (pCEP4)	RNA polymerase II	KELLY/stable transfection of insertless vector (pCEP4)	IgG
Cc4	KELLY/stable PLZF overexpression(PLZF-C-HA/pCEP4)	PLZF	KELLY/stable PLZF overexpression(PLZF-C-HA/pCEP4)	IgG
Cc5	KELLY/stable transfection ofinsertless vector (pCEP4)	PLZF	KELLY/stable transfection of insertless vector (pCEP4)	IgG
Cc6	HEK293T/stable PLZFoverexpression (PLZF/pCEP4)	PLZF	HEK293T/stable PLZF overexpression (pCEP4)	PLZF

Each two-color tiling array was hybridized with the amplified DNA of two experimental conditions (intervention 1 and intervention 2) simultaneously. The used antibodies for the DNA enrichments are indicated.

### ChIP-chip Experiments

To validate our microarray findings, we performed a set of ChIP-chip experiments under similar experimental conditions and respective controls as the microarrays presented above (Table 4). Since pol II is a prerequisite for mRNA transcription [30], [31], we initially focused our Chip-chip analyses on pol II recruitment (Cc1, Cc2, Cc3).

**Figure 5:**
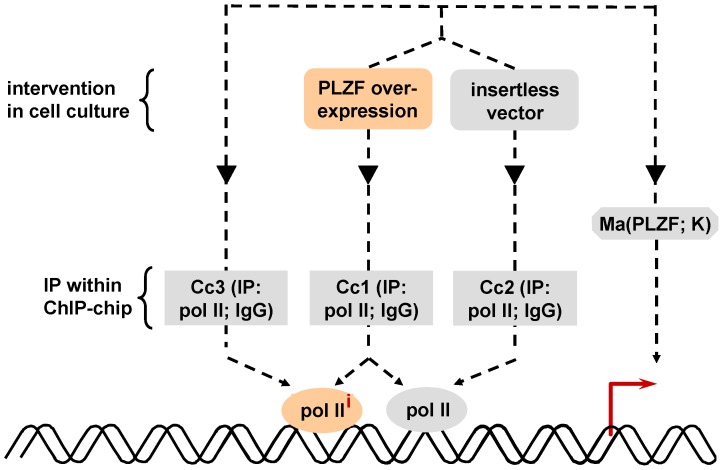
Experimental scheme of the ChIP-chips Cc1, Cc2 and Cc3 in combination with microarray Ma(PLZF; K). Pol II: RNA polymerase II binding sites in cells without intervention; pol II^i^: additional RNA polymerase II binding sites induced by PLZF overexpression.

**Figure 6:**
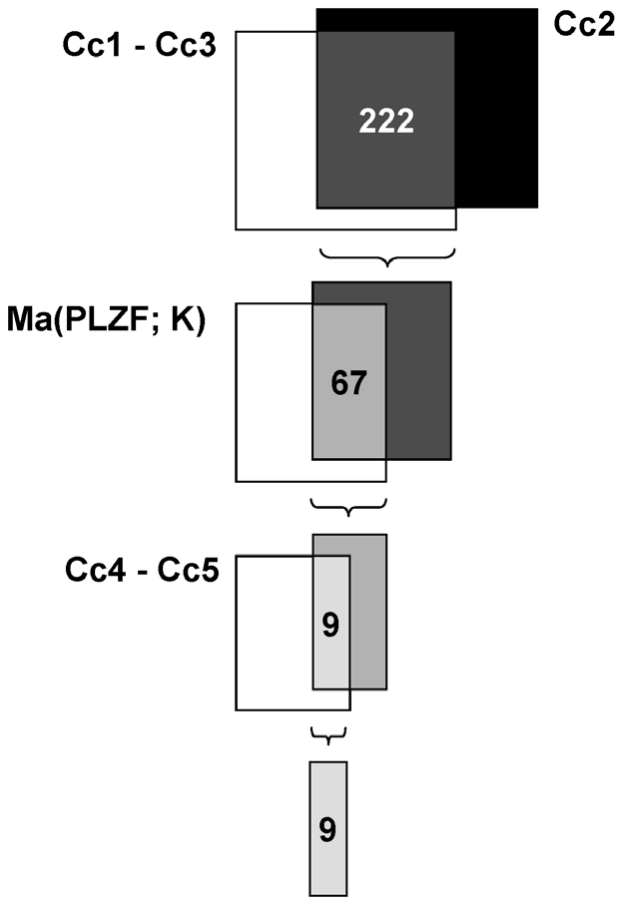
Sequential algorithm to identify target genes common to all interventions. A cascade of intersections of ChIP-chip and microarray results were performed as indicated yielding nine genes with an enhanced recruitment of PLZF and pol II as well as concurrent increased mRNA levels. The respective denotations of these nine genes are given in Table 5.

The initial ChIP-chip experiment (Cc1) was realised in KELLY cells overexpressing PLZF (Figure 5). Cc1 identifies basal (i.e., without effect of PLZF overexpression) and PLZF-induced pol II recruitment. Cc3 identifies basal pol II recruitment, whereas Cc2 is a measure of PLZF-induced pol II recruitment (Figure 5). Therefore, genes on which pol II recruitment were induced by PLZF overexpression are given by experiment Cc2 but also by the subtraction of Cc3 results from Cc1 results. Regarding Cc1, a total of 2791 unique genes were found to recruit pol II in their core promoter regions as defined from nucleotide position -112 to +77 relative to the TSS using the Genomeinspector and Regionminer software (Genomatix). 61 and 1488 unique genes were detected in Cc3 and Cc2, respectively. The generated intersection of Cc2 and the relative complement of Cc3 in Cc1 (i.e., Cc1– Cc3) resulted in 222 unique genes (Figure 6). Afterwards, this data list was compared to the corresponding microarray Ma(PLZF; K) which yielded 67 overlapping genes. Consistent with an increased pol II recruitment, 66 of these 67 overlapping genes showed an elevated mRNA level. The top ten overrepresented transcripts from these 66 genes are shown in Table S11 in File S1. IPA analysis demonstrated that the 67 transcript were significantly (p<0.05) associated with diabetes, atherosclerosis and mood disorders (data not shown).

**Figure 7:**
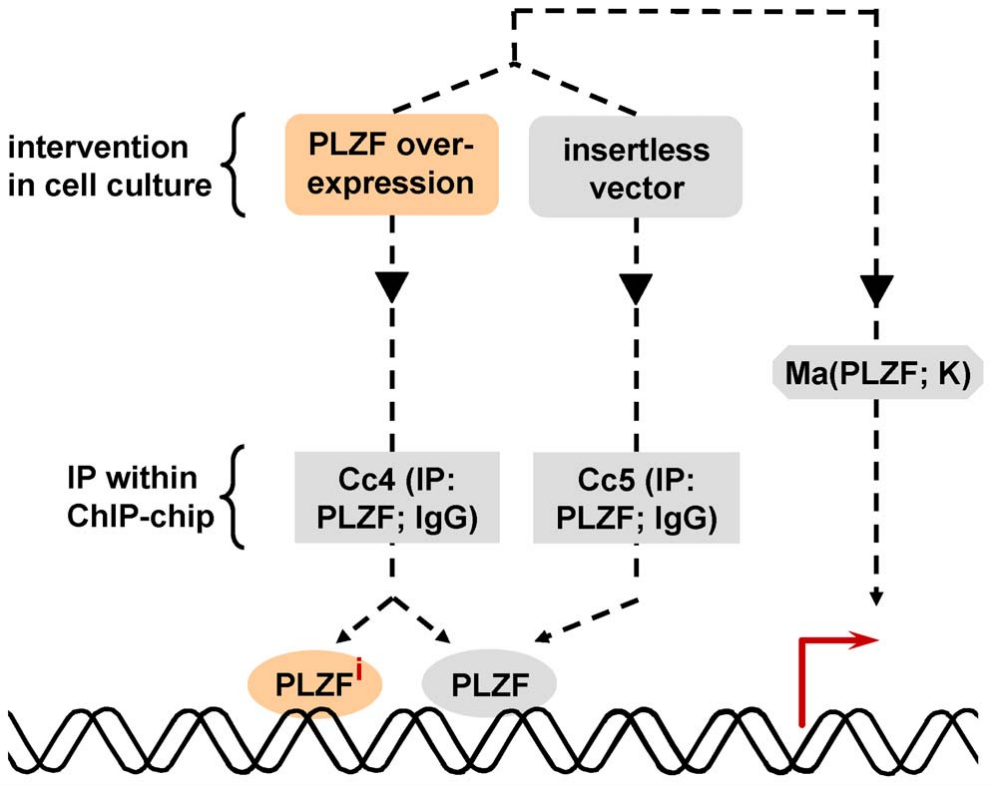
Experimental scheme of the ChIP-chips of Cc4 and Cc5 in combination with the microarray Ma(PLZF; K). PLZF: PLZF binding sites in cells without intervention; PLZF^i^: additional PLZF binding sites (i.e., recruitment of endogeneous and overexpressed PLZF on the same *cis*-element) induced by PLZF overexpression.

The ChIP-chip experiment Cc4 was performed in KELLY cells overexpressing PLZF (Table 4, Figure 7). Analogous to the previous pol II ChIP-chip experiments, Cc4 identifies basal (i.e., without effect of PLZF overexpression) and concurrent PLZF-induced PLZF recruitment whereas Cc5 identifies basal PLZF recruitment (Table 4, Figure 7). Therefore, genes on which PLZF recruitment was induced by PLZF overexpression were given by subtraction of experiment Cc5 results from Cc4 results.

A total of 5502 regions - corresponding to 1213 unique genes - were found to recruit PLZF in experiment Cc4. 337 of these genes showed at least one PLZF binding site as defined by Genomatix using Regionminer software (Genomatix). Concerning Cc5, in total 4004 regions were detected corresponding to 828 unique genes. 241 genes contained a PLZF *cis*-element.

The generated relative complement of Cc5 in Cc4 (i.e., Cc4– Cc5) resulted in 5328 regions and 1107 unique genes of which 303 contained at least one PLZF binding site.

Subsequently, this gene list was intersected with the 66 genes derived from the previous section (i.e., genes with enhanced pol II recruitment and a concurrent increased mRNA level) resulting in nine overlapping genes (Table 5 and Figure 6).

**Table 5:** List of genes on which pol II as well as PLZF were recruited by PLZF overexpression and which additionally exhibited an increased mRNA level induced by PLZF overexpression (Cc1\Cc3 ∩ Cc2 ∩ Cc4\Cc5 ∩ Ma(PLZF; K).

gene ID	gene symbol	description	fold changeMa(PLZF; K)	Cc6	PLZF *cis*-element
6373	CXCL11	chemokine (C-X-C motif) ligand 11	3.58	✓	✓
22872	SEC31A	SEC31 homolog A (*S. cerevisiae*)	2.62	–	–
10827	FAM114A2	family with sequence similarity 114, member A2	2.53	✓	✓
64208	POPDC3	popeye domain containing 3	2.22	–	–
158427	TSTD2	thiosulfate sulfurtransferase (rhodanese)-like domain containing 2	2.19	✓	✓
55729	ATF7IP	activating transcription factor 7 interacting protein	2.17	–	–
51175	TUBE1	tubulin, epsilon 1	2.17	✓	✓
261729	STEAP2	six transmembrane epithelial antigen of the prostate 2	2.17	✓	✓
79813	EHMT1	euchromatic histone-lysine N-methyltransferase 1	2.13	–	–

The presence of the respective genes in the Cc6 results dataset and the presence of PLZF cis-elements within the ChIP-enriched regions are indicated by checkmarks.

To further validate our findings, these genes were intersected with Cc6 results, which based on an IP against PLZF in HEK293T cells, yielding in five genes (Table 5). PLZF *cis*-elements were identified in all of them (Table 5).

Finally, all genes given in Table 5 and selected additional transcript (e.g., based on consistency with the literature as highlighted in Tables S2, S3 and S5 to S11 in File S1) were subjected to real-time PCR analyses for putative validation (Table 6). 15 of 25 microarray results could be confirmed by real-time PCRs; regarding CXCL11 and STEAP2 no amplification products were obtainable.

**Table 6:** Validation of microarray- and ChIP-chip-derived results by real-time PCR.

			Ma(si(P)RR)								Ma(bafi)					
			microarray		validation by PCR						microarray	validation by PCR				
gene symbol	gene ID		rel. fold change[%]		SER[%]			n	SEM	p-value	rel. fold change[%]	SER[%]		n	SEM	p-value
ADORA2A	135		**31.53**		**16.97**			**3**	**1.71**	**0.012**	**184.68**	**624.55**		**3**	**44.74**	**0.000**
CRH	1392		**30.15**		**4.87**			**3**	**0.82**	**0.001**	–	–			–	–
CTSB	1508		–		–			–	–	–	167.02	110.37		3	7.36	0.402
FN1	2335		164.72		100.38			6	15.14	0.982	**171.71**	**165.38**		**3**	**25.64**	**0.072**
ID1	3397		**196.79**		**166.05**			**6**	**14.77**	**0.002**	–	–		–	–	–
ID3	3399		–		–			–	–	–	62.94	157.32		3	6.55	0.000
IGF2	3481		**41.68**		**42.75**			**6**	**4.03**	**5.27^.^ 10^−6^**	64.06	131.35		3	13.45	0.113
NOX4	50507		174.11		128.81			6	35.11	0.103	–	–		–	–	–
PGK1	5230		**33.80**		**36.50**			**3**	**1.35**	**0.001**	–	–		–	–	–
POPDC3	64208		–		–			–	–	–	170.53	90.35		3	10.22	0.406
SEC31A	22872		–		–			–	–	–	**164.55**	**222.04**		**3**	**27.83**	**0.013**
TUBE1	51175		**191.85**		**148.49**			**3**	**9.74**	**0.010**	–	–		–	–	–
				**Ma(PLZF; K)**							**Ma(PLZF; H)**					
				**microarray**		**validation by PCR**					**microarray**	**validation by PCR**				
**gene symbol**		**gene ID**		**rel. fold change** **[%]**		**SER** **[%]**	**n**		**SEM**	**p-value**	**rel. fold change** **[%]**	**SER** **[%]**	**n**		**SEM**	**p-value**
ADORA2A		135		**40.33**		**29.25**	**3**		**1.91**	**0.009**	–	–			–	
ATF7IP		55729		212.87		95.77	6		2.03	0.561	–	–			–	
CRH		1392		**34.87**		**33.23**	**6**		**2.53**	**0.000**	–	–			–	
EHMT1a		79813		217.35		80.61	5		5.84	0.069	–	–			–	
FAM113A2		10827		253.31		87.42	6		7.95	0.178	–	–			–	
FN1		2335		–		–			–		179.99	64.29	9		6.87	0.005
ID1		3397		**40.33**		**14.19**	**6**		**1.34**	**3.73^.^ 10^−6^**	–	–			–	
ID2		3398		**43.53**		**32.67**	**3**		**4.65**	**0.006**	–	–			–	
ID3		3399		**50.00**		**32.39**	**6**		**5.10**	**2.30^.^ 10^−6^**	**179.75**	**684.28**	**3**		**39.78**	**0.000**
IGF2		3481		–		–			–		185.75	96.31	9		10.03	0.804
NOX4		50507		**224.79**		**268.56**	**6**		**43.70**	**0.005**	–	–			–	
POPDC3		64208		221.91		106.41	6		8.23	0.522	–	–			–	
SEC31A		22872		**262.08**		**140.07**	**3**		**7.03**	**0.006**	–	–			–	
TSTD2		158427		218.86		59.17	6		14.50	0.034	–	–			–	
TUBE1		51175		**216.94**		**162.25**	**3**		**23.57**	**0.072**	50.44	47.67	3		2.31	0.048

Total RNA used for microarray analyses was subjected to real-time PCR quantification. The standardised expression ratio (SER) indicates the expression normalised to 18S rRNA and standardised to control condition (scrambled siRNA or vehicle). A SER of 100% indicates no expression difference versus control. P-values were based on a two-tailed, unpaired t-test. SEM: standard error of the mean with respect to the intervention; n: number of single PCR measurements. Fold changes are given in percent relative to the control condition based on the microarray data. Validated Genes, i.e., those which showed significantly (p<0.05) altered mRNA levels as detected by real-time PCR and which additionally are concordantly regulated in microarray-analyses, are highlighted in bold.

## Discussion

In this study, several different interventions have been used to dissect the distinct signal transduction pathways downstream of the (P)RR (Figure 1). Genome-wide expression analysis revealed transcript clusters commonly regulated by all molecular functions (i.e., V-ATPase-mediated and PLZF-mediated) of the (P)RR (Figure 3). Moreover, the fact that the majority of regulated transcripts is not part of the intersections of the different interventions (Figure 3) clearly indicates that the (P)RR exerts biological functions via distinct pathways.

Since a soluble isoform has been described [32] and since the (P)RR is a crucial adapter protein of the Wnt pathway [16], there are probably even more distinct pathways and related sub-transcriptomes downstream of the (P)RR. Moreover, the effects of renin and prorenin on these distinct sub-pathways remain to be elucidated. Nevertheless, combining interventions interfering with all these sub-pathways by a microarray approach is beyond the scope of this publication.

In this analysis, several genes regulated upon genistein treatment, which do not overlap with (P)RR-PLZF/V-ATPase function, were identified (Table 3 and Figure 3). This result is not unexpected because genistein is known to have pleiotropic effects. It can interact with estrogen receptors alpha and beta [33], [34]. Furthermore, genistein is an inhibitor of tyrosine kinases [35] and also mediates effects via NFkappaB [36] and Smad [37] signal transductions.

The inhibition of nuclear translocation of PLZF by Genistein [7] is a likely explanation for the concordantly regulated genes by siRNA((P)RR), genistein and bafilomycin interventions (Table 3 and Figure 3). In this context, it is interesting to note that 15% of all genes regulated by genistein were also regulated by bafilomycin (Table 3).

Nevertheless, overtly opposite interventions (genistein treatment and PLZF overexpression) caused a concordant regulation of 20 transcripts (Table 3, Table S9 in File S1). This might be linked to the pleiotropic effects of genistein discussed above and/or by the PLZF overexpression putatively associated with off-target effects.

Focusing on certain target genes, our microarray analyses demonstrated a downregulation of the (P)RR transcript after PLZF overexpression in human neuronal KELLY cells which is in agreement with our previous work that PLZF is a repressor of the (P)RR promoter in non-neuronal cells [5], [6]. Furthermore, PLZF overexpression caused a repression of several other V-ATPase family members, implying a coregulation of this molecular machine.

As recently discussed, MAPK are well-known direct and/or indirect downstream targets of the (P)RR [3]. IPA analysis of microarray data revealed MAPK associated molecules after silencing of (P)RR using siRNA (Table 2). Consistent with our ChIP-chip analyses, recruitment of pol II was detected on genes such as MAPK1, MAPK6, MAPK9, MAPK10, MAPKAP1 (mitogen-activated protein kinase associated protein 1), LAMPTOR2 (late endosomal/lysosomal adaptor, MAPK and MTOR activator 2), and JKAMP (JNK1/MAPK8-associated membrane protein) (data not shown). In addition, PLZF enrichment could be demonstrated on, for example, MAPKAPK2 (mitogen-activated protein kinase-activated protein kinase 2), MAP3K1 (mitogen-activated protein kinase kinase kinase 1) and MAP3K2 genes (data not shown).

The mannose 6-phosphate/insulin-like growth factor II (M6P/IGF-II) receptor (IGF2R or IGF-IIR) is a clearance receptor of renin and prorenin [38] and can also bind IGF-II [39]. Here, we were able to show by real-time PCR that the (P)RR positively regulates the IGF2.

Consistent with Saris et al. [23], we observed that an inhibition of (P)RR activity by siRNA and also bafilomycin A1 *increased* mRNA of fibronectin (Tables S2 and S3 in File S1). The latter could be validated by real-time PCR (Table 6). In contrast, renin and prorenin are known to induce fibronectin expression via the (P)RR [40], [41], [42]. Fibronectin is an important component of the extracellular matrix and is dysregulated in fibrotic disease conditions as well as in tumorigenesis [43]. Nevertheless, the importance of fibronectin as a mediator of these pathophysiologies seems to be cell type-specific - or even dependent on the constitutive versus ligand-mediated activity of the (P)RR [unpublished data] - because this receptor can positively and negatively regulate fibronectin expression as discussed above. The importance of cell type specificities is further supported by the regulation of Nox4, a part of the NADPH oxidase complex. In HEK cells this transcript was positively regulated by (P)RR activation [41] whereas in KELLY cells Nox4 mRNA levels are negatively regulated by this receptor but positively regulated by PLZF overexpression as indicated by real-time PCR (Table 6) Concerning further genes relevant for the pathogenesis of fibrosis, we were able to confirm the observation of He et al. [44] that (P)RR represses MMP-2 expression (Table S2 in File S1). In Ma(PLZF, H) a fold change of 1.94 was observed concerning MMP-2 expression indicating an upregulation by PLZF (data not shown). In apparent contrast to HEK cells, in which siRNA against the (P)RR decreased collagen 4 on protein level [44], we observed increased mRNA levels of a set of collagen types (3, 4, 6, 7, 9, 11, 12, 14, 15, 24 and 27) in neuronal cells by this intervention.

In this study we were able to show that PLZF overexpression causes an increased expression of the transcription factor GATA-4 (Tables S6 and S7 in File S1). Accordingly, a recent publication was able to demonstrate that PLZF can increase GATA-4 gene expression downstream of angotensin II in the context of cardiac hypertrophy [45]. Moreover, GATA-4 is found in the developing CNS and can inhibit the proliferation of astrocytes [46].

Inhibitor of DNA binding (ID) genes can block cellular differentiation and can exert pro-proliferative effects [47]. They contribute to mammalian nervous system development [48] and are dysregulated in human tumours [47]. ID1 and ID3 were shown to be upregulated in PLZF-overexpressing Jurkat cells [29] and transduction of PLZF in myeloid progenitor cells increased ID2 mRNA [49]. In our study, overexpression of PLZF was associated with increased and decreased transcript levels of ID genes in neuronal and epithelial cells, respectively (Tables S6 and S7 in File S1). Importantly, the regulation of ID genes in the context of (P)RR signal transduction cascade could be validated by real-time PCR (Table 6). Furthermore, our bioinformatic network analyses indicated that PLZF overexpression was linked with transcriptional signatures associated with brain development and tumorigenesis (Table 2).

Also in the context of PLZF-mediated gene regulation, cell type specificities seem to be relevant. In myeloid progenitor cells PLZF activates myc mRNA expression [49] whereas PLZF represses myc mRNA in embryonic fibroblasts [50]. We could demonstrate that the myc related gene MYCN was upregulated in epithelial cells by PLZF overexpression.

Several publications using conditional knockout approaches have demonstrated the crucial role of the (P)RR gene concerning cardiac [18] and renal development [17], [51]. Concerning bone development, V-ATPases are essential for bone resorption by osteoclasts which is tightly linked to matrix formation by osteoblasts [52]. Furthermore, bafilomycin derivatives have been successfully tested regarding osteoporosis in an animal model [53]. In addition, PLZF seems to be involved in early osteoblastic differentiation [54]. With respect to the nervous system, PLZF is expressed in temporally dynamic and spatially restricted patterns during brain development [55]. With respect to (P)RR, a mutation of this gene can cause a X-linked mental retardation and epilepsy syndrome in humans [56]. Consistently, a zebrafish (P)RR mutant displays a reduced head size as well as a central nervous system necrosis [57]. All of these observations are in full agreement with our IPA analyses (Table 2) showing the role of the (P)RR-PLZF cascade in different developmental processes.

Table S12 in File S1 gives individual genes underlying the transcriptional clusters involved in brain development (Table 2). Interestingly, four transcripts (DCX [58], ATRX [59], MECP2 [60] and NIPBL [61]) regulated under siRNA against (P)RR have also been associated with mental retardation and/or epilepsy (Table S12 in File S1). Moreover, the genes RELN [58] and SIM1 [62], regulated by PLZF overexpression, are involved in epilepsy and mental retardation, respectively (Table S12 in File S1). Therefore, these six genes might represent putative downstream candidate genes in patients with a (P)RR mutation mentioned above.

In the context of V-ATPase and development it is of interest to note that our intervention using bafilomycin identified lysosomal-associated membrane protein 2 (LAMP2) as being upregulated (Table S3 in File S1). Consistently, (P)RR-depleted cardiomyocytes [18], glomeruli [51] and podocytes [63] exhibit multivesicular vacuoles with enriched LAMP2.

Our bioinformatic data also indicate that the (P)RR might be a host factor regarding HIV (Table 2). Consistently, there is experimental evidence that (P)RR/ATP6AP2 is involved in influenza virus infection [12], [64]. This implies that small molecule-based drugs inhibiting the (P)RR pathway, which are currently developed regarding cardiovascular endorgan-damage [3], might have an additional antiviral indication. However, we are aware that cell culture experiments are the base of our experiments and that the respective translation to *in vivo* conditions is speculative.

In this study a ChIP-chip approach was used to validate our microarray data (Table 4). Promoter regions, on which pol II recruitment was induced by PLZF overexpression, were identified using an anti-pol II immunoprecipitation. The resulting set of 222 unique genes was combined with the microarray-derived transcripts which were upregulated in PLZF overexpressing cells. The resulting 66 genes showed similar pathophysiological network associations as revealed by our microarray analyses (diabetes mellitus, atherosclerosis and mood disorder; Table 2 and Table S4 in File S1).

Subsequently, these 66 genes were analysed regarding PLZF recruitment by anti-PLZF immunoprecipitation yielding nine genes, all of which were characterised by a more than two-fold increase in mRNA expression levels (Table 5). Five of these nine genes were also identified in ChIP-chip experiment Cc6 (based on untagged PLZF overexpression in HEK293T cells; Table 4) indicating independence of cell type and of presence or absence of a tag. Strikingly, four of these genes harbour at least one PLZF *cis*-element indicating a direct regulation by this transcription factor.

The indirect recruitment of transcription factors to non-consensus sites via interaction with other direct DNA-binding transcription factors is a known phenomenon [65], [66]. Also in this study, the enrichment of several other *cis*-elements was identified in our ChIP-chip experiments. For example, enrichment of E2F and GATA motifs was identified in anti-PLZF ChIP-chip experiments (Cc4 and Cc5) using the *cis*-regulatory Element Annotation System (CEAS) [67] and the Chipinspector software (data not shown). PLZF, retinoblastoma protein (pRb) and E2 promoter-binding factor-1 (E2F-1) are known to form a regulatory complex [68] suggesting that PLZF might be tethered by E2F *cis*-elements. Furthermore, PLZF can directly interact with GATA-2 [69] implying a similar tethering mechanism. Regarding one major finding of our study, the identification of nine genes with intersected microarray and ChIP-chip data, SEC31a and TUBE1 are of special interest due to their validation by real-time PCR analyses.

Finally, to better assess the general relevance of all individual genes mentioned in this study, a table based on the Eldorado database summarising the biological and pathophysiological functions of the respective transcripts is given (Table S13 in File S1).

The (P)RR is a novel pharmacological target regarding cardiorenal end-organ damage [3] and cancer [16]. The transcriptional signatures downstream of certain components of the (P)RR signal transduction cascade identified in this study might, therefore, be useful in drug discovery to assess the specificity or pleiotropy of developmental compounds. By comparing microarray data of drug candidates (i.e., hits or leads) with the results obtained here, it would be possible to select compounds which cause for example a transcriptional signature similar to an inhibition of (P)RR’s signal transduction but without affecting the V-ATPase-associated function of this receptor (or *vice versa*). This so-called magic shotgun approach is thought to increase the effectiveness and to limit putative side effects of future drugs in general [70].

Furthermore, certain transcripts such as adenosine A2a receptor (ADORA2A) and corticotropin releasing hormone (CRH) (Table S2 in File S1) with a high degree of regulation (i.e., a high fold change value) have been identified and also validated (Table 6) within this study. These might serve as putative pharmacodynamic biomarkers in proof-of-mechanism (animal) studies of drug candidates after RNA isolation from tissues and/or blood cells [71].

In conclusion, we were able to identify distinct and overlapping genetic signatures as well as novel target genes downstream of the different molecular functions (Figure 1) of the (P)RR. Moreover, IPA analyses of our data confirm the role of (P)RŔs signal transduction pathways in cardiovascular disease and tumorigenesis highlighting its role as a pharmacological target.

## Supporting Information

File S1 **Supporting Tables.**
(DOC)
