# Supplementary material for: Distinct Signal Transduction Pathways Downstream of the (P)RR Revealed by Microarray and ChIP-chip Analyses
Source: PLoS One. 2013 Mar 4;8(3):e57674. doi: 10.1371/journal.pone.0057674 (PMC3587649; doi:10.1371/journal.pone.0057674)
Supplement: File S1 — Supporting Tables. (DOC) [file pone.0057674.s001.doc]

Table S1. List of primer pairs used for microarray data validation by real-time PCR.

| **gene symbol** | **sense** | **antisense** |
| --- | --- | --- |
| ADORA2 | 5'-TCTTCAGTCTCCTGGCCATC-3' | 5'- AGTGGTTCTTGCCCTCCTTT-3' |
| ATF7IP | 5'- TGGACTAACCCTGGGATCAA-3' | 5'- TGCCATTTTGACTCTGAACG-3' |
| CRH | 5’- ACCGCCAACTTTTTCCGCGTG-3’ | 5’- TCGGCCCTGGCCATTTCCAAGA-3’ |
| CTSB | 5’- AATGCGCACGTCAGCGTGGA-3’ | 5’- AGCCGTTGACGTGGTGCTCA-3’ |
| EHMT1 | 5'- ATTCCACTGTGGGTTCCAAG-3' | 5'- TGGAAGTCCTGCTGTCCTCT-3' |
| FAM113A2 | 5’- ATTGGGGGAGCTGGGGCAAGT-3’ | 5’- TGGGACCAGGGATTCCAAGGGA-3’ |
| FN1 | 5'- CATGAAGGGGGTCAGTCCTA-3' | 5'-CCTTCTCCCAGGCAAGTACA-3' |
| ID1 | 5’- AGGTGGTGCGCTGTCTGTCTGA-3’ | 5’- CCACCTTGCTCACCTTGCGGTT-3’ |
| ID2 | 5’- TCTTGGACCTGCAGATCGCCCT-3’ | 5’- GCCACACAGTGCTTTGCTGTCA-3’ |
| ID3 | 5’- CGCTGAGCTTGCTGGACGACAT-3’ | 5’- AGCTCGGCTGTCTGGATGGGAA-3’ |
| IGF2 | 5’- CAAGCCGTGTGAGCCGTCGC-3’ | 5’- TGTCCGGAAGCACGGTCGGA-3’ |
| NOX4 | 5'- TTGGCTTTGGATTTCTGGAC-3' | 5'- CATTGTGAGGGTAAATGGATGA-3' |
| PGK1 | 5’- GACGCTGGACAAGCTGGACGTT-3’ | 5’- TTGTCAGGCATGGGCACACCA-3’ |
| POPDC3 | 5'- TGGCGAATTTCTGCATTACA-3' | 5'- AAAAAGGCGGGAGATGTAGC-3' |
| SEC31A | 5'- TCAGCAATTGGATGCAACAT-3' | 5'- CACCTGCAATCAGAACTCCA-3' |
| TSTD2 | 5’- GACCAAGGAGATGACCTGG -3’ | 5’- GATGCTGGTTTGGTCTGTGA-3’ |
| TUBE1 | 5’- GGATCTGGACTTGGCACATT-3’ | 5’- ACCATGAGGTCGATTTTGCT-3’ |
|  |  |  |

Table S2. Transcriptome analysis of KELLY cells after repression of the (P)RR by siRNA (Ma(si(P)RR)). The 10 genes with the highest and the 10 genes with the lowest fold changes (relative to a scrambled control siRNA) are shown. Fold changes > 1 (< 1) indicate increased (decreased) mRNA levels. Hypothetical and functionally uncharacterized genes are not given. The gene encoding (P)RR (ATP6AP2) is highlighted in bold. Additionally, target genes previously published are highlighted in italics.

| **gene ID** | **gene symbol** | **description** | **fold change Ma(si(P)RR)** |
| --- | --- | --- | --- |
| 4199 | ME1 | malic enzyme 1, NADP(+)-dependent, cytosolic | 3.63 |
| 10026 | PIGK | phosphatidylinositol glycan anchor biosynthesis, class K | 2.89 |
| 9818 | NUPL1 | nucleoporin like 1 | 2.71 |
| 116969 | ART5 | ADP-ribosyltransferase 5 | 2.57 |
| 81616 | ACSBG2 | acyl-CoA synthetase bubblegum family member 2 | 2.55 |
| 3400 | ID4 | inhibitor of DNA binding 4, dominant negative helix-loop-helix protein | 2.46 |
| 57580 | PREX1 | phosphatidylinositol-3,4,5-tr-isphosphate-dependent Rac exchange factor 1 | 2.46 |
| 7409 | VAV1 | vav 1 guanine nucleotide exchange factor | 2.45 |
| 79786 | KLHL36 | kelch-like 36 (Drosophila) | 2.43 |
| 10140 | TOB1 | transducer of ERBB2, 1 | 2.43 |
| … | … | … | … |
| *6446* | *SGK1* | *serum/glucocorticoid regulated kinase 1* | *2.39*  *[[[1]](#endnote-2)]* |
| *10769* | *PLK2* | *polo-like kinase 2* | *2.25 [Error: Reference source not found]* |
| *5168* | *ENPP2* | *ectonucleotide pyrophosphatase/phosphodiesterase 2* | *1.89 [Error: Reference source not found]* |
| *6659* | *SOX4* | *SRY (sex determining region Y)-box 4* | *1.89* *[Error: Reference source not found]* |
| *6498* | *SKIL* | *SKI-like oncogene* | *1.87 [Error: Reference source not found]* |
| *9771* | *RAPGEF5* | *Rap guanine nucleotide exchange factor (GEF) 5* | *1.78 [Error: Reference source not found]* |
| *1285* | *COL4A3* | *collagen, type IV, alpha 3 (Goodpasture antigen)* | *1.77* *[[[2]](#endnote-3)]* |
| *4313* | *MMP2* | *matrix metallopeptidase 2 (gelatinase A, 72kDa gelatinase, 72kDa type IV collagenase)* | *1.77 [Error: Reference source not found]* |
| *1356* | *CP* | *ceruloplasmin (ferroxidase)* | *1.74 [Error: Reference source not found]* |
| *50507* | *NOX4* | *NADPH oxidase 4* | *1.74* *[[[3]](#endnote-4)]* |
| *4092* | *SMAD7* | *SMAD family member 7* | *1.71 [Error: Reference source not found]* |
| *2335* | *FN1* | *fibronectin 1* | *1.65* *[[[4]](#endnote-5)]* |
| *1286* | *COL4A4* | *collagen, type IV, alpha 4* | *1.64 [1]* |
| 1392 | CRH | corticotropin releasing hormone | 0.30 |
| 135 | ADORA2A | adenosine A2a receptor | 0.31 |
| **10159** | **ATP6AP2** | **ATPase, H+ transporting, lysosomal accessory protein 2** | **0.33** |
| 9791 | PTDSS1 | phosphatidylserine synthase 1 | 0.34 |
| 5230 | PGK1 | phosphoglycerate kinase 1 | 0.34 |
| 10007 | GNPDA1 | glucosamine-6-phosphate deaminase 1 | 0.35 |
| 9607 | CARTPT | CART prepropeptide | 0.36 |
| 729041 | LOC729041 | fatty-acid amide hydrolase 1-like | 0.36 |
| 83643 | CCDC3 | coiled-coil domain containing 3 | 0.37 |
| 1389 | CREBL2 | cAMP responsive element binding protein-like 2 | 0.38 |

Table S3. Transcriptome analysis of KELLY cells after incubation with bafilomycin A1 (Ma(bafi)). The 10 genes with the highest and the 10 genes with the lowest fold changes (relative to a DMSO control) are shown. Fold changes > 1 (< 1) indicate increased (decreased) mRNA levels. Hypothetical and functionally uncharacterized genes are not given. Additionally, target genes previously published are highlighted in italics.

| **gene ID** | **gene symbol** | **description** | **fold change Ma(bafi)** |
| --- | --- | --- | --- |
| 85028 | SNHG12 | small nucleolar RNA host gene 12 (non-protein coding) | 2.68 |
| 692073 | SNORA16A | small nucleolar RNA, H/ACA box 16A | 2.66 |
| 6284 | S100A13 | S100 calcium binding protein A13 | 2.41 |
| 9512 | PMPCB | peptidase (mitochondrial processing) beta | 2.31 |
| 134266 | GRPEL2 | GrpE-like 2, mitochondrial (E. coli) | 2.23 |
| 26003 | GORASP2 | golgi reassembly stacking protein 2, 55kDa | 2.22 |
| 55839 | CENPN | centromere protein N | 2.20 |
| 79714 | CCDC51 | coiled-coil domain containing 51 | 2.19 |
| 10009 | ZBTB33 | zinc finger and BTB domain containing 33 | 2.17 |
| 5716 | PSMD10 | proteasome (prosome, macropain) 26S subunit, non-ATPase, 10 | 2.16 |
| … | … | … | … |
| *3920* | *LAMP2* | *lysosomal-associated membrane protein 2* | *1.77 [[[5]](#endnote-6), [[6]](#endnote-7)]* |
| 677813 | SNORA30 | small nucleolar RNA, H/ACA box 30 | 0.38 |
| 1616 | DAXX | death-domain associated protein | 0.39 |
| 594839 | SNORA33 | small nucleolar RNA, H/ACA box 33 | 0.40 |
| 1303 | COL12A1 | collagen, type XII, alpha 1 | 0.41 |
| 677775 | SCARNA5 | small Cajal body-specific RNA 5 | 0.41 |
| 692225 | SNORD94 | small nucleolar RNA, C/D box 94 | 0.42 |
| 116969 | ART5 | ADP-ribosyltransferase 5 | 0.43 |
| 54487 | DGCR8 | DiGeorge syndrome critical region gene 8 | 0.44 |
| 7634 | ZNF80 | zinc finger protein 80 | 0.45 |
| 10652 | YKT6 | YKT6 v-SNARE homolog (S. cerevisiae) | 0.45 |

Table S4. IPA analyses of intersected microarray data from experiment Ma(si(P)RR), Ma(bafi), Ma(PLZF; H, K), Ma(geni). The significance levels, the number of involved molecules (n) as well as their percentages relative to the input datasets are given. The functional classification of a specific gene can be redundant due to the assignment of one gene to more than one category.

|  | **Ma(si(P)RR) /** | | | **Ma(si(P)PR) /** | | | **Ma(bafi) /** | | | **Ma(bafi) /** | | | **Ma(si(P)RR) /** | | |
| --- | --- | --- | --- | --- | --- | --- | --- | --- | --- | --- | --- | --- | --- | --- | --- |
|  | **Ma(bafi)** | | | **Ma(PLZF; H, K)** | | | **Ma(PLZF; H, K)** | | | **Ma(geni)** | | | **Ma(geni)** | | |
|  | input: |  | 238 | input |  | 44 | input |  | 46 | input |  | 95 | input |  | 76 |
| **function annotation** | **p-value** | **n** | **[%]** | **p-value** | **n** | **[%]** | **p-value** | **n** | **[%]** | **p-value** | **n** | **[%]** | **p-value** | **n** | **[%]** |
| tumorigenesis | 6.9E-03 | 66 | 28 | -- | -- | -- | -- | -- | -- | -- | -- | -- | -- | -- | -- |
| *gastrointestinal tract cancer* | 3.6E-04 | 26 | 11 | -- | -- | -- | -- | -- | -- | -- | -- | -- | -- | -- | -- |
| *colorectal cancer* | 3.4E-04 | 24 | 10 | -- | -- | -- | -- | -- | -- | -- | -- | -- | 5.2E-03 | 9 | 12 |
| diabetes mellitus | 5.1E-07 | 58 | 24 | 4.1E-04 | 16 | 36 | -- | -- | -- | 3.7E-04 | 21 | 22 | -- | -- | -- |
| tissue development | 1.7E-03 | 46 | 19 | 5.0E-06 | 19 | 43 | 1.5E-03 | 15 | 33 | -- | -- | -- | 1.9E-03 | 18 | 24 |
| *development of connective tissue* | 1.3E-03 | 18 | 8 | -- | -- | -- | -- | -- | -- | -- | -- | -- | -- | -- | -- |
| *development of bone* | 2.0E-03 | 16 | 7 | -- | -- | -- | -- | -- | -- | -- | -- | -- | -- | -- | -- |
| atherosclerosis | 2.3E-06 | 37 | 16 | -- | -- | -- | 7.4E-04 | 11 | 24 | 1.9E-03 | 13 | 14 | 1.2E-07 | 19 | 25 |
| *coronary artery disease* | 4.0E-06 | 34 | 14 | 9.2E-06 | 13 | 30 | 1.3E-03 | 10 | 22 | 6.9E-03 | 11 | 12 | 7.6E-05 | 14 | 18 |
| rheumatoid arthritis | 3.7E-05 | 39 | 16 | 9.6E-03 | 10 | 23 | -- | -- | -- | 4.7E-04 | 16 | 17 | 5.0E-03 | 13 | 17 |
| inflammatory bowel disease | 8.7E-08 | 38 | 16 | -- | -- | -- | -- | -- | -- | -- | -- | -- | -- | -- | -- |
| bipolar disorder | -- | -- | -- | -- | -- | -- | -- | -- | -- | 2.6E-04 | 13 | 14 | 5.2E-03 | 10 | 13 |
| hypertension | 3.0E-03 | 24 | 10 | -- | -- | -- | -- | -- | -- | -- | -- | -- | -- | -- | -- |
| Alzheimer's disease | -- | -- | -- | -- | -- | -- | -- | -- | -- | -- | -- | -- | 2.0E-03 | 10 | 13 |

Table S5. Transcripts with the highest and lowest fold changes within the intersection of Ma(si(P)RR) and Ma(bafi). Fold changes > 1 (< 1) indicate increased (decreased) mRNA levels. Ranking is based on Ma(si(P)RR) results. Hypothetical and functionally uncharacterized genes are not given.

| **gene ID** | **gene symbol** | **description** | **fold change Ma(si(P)RR)** | **fold change Ma(bafi)** |
| --- | --- | --- | --- | --- |
| 23043 | TNIK | TRAF2 and NCK interacting kinase | 2.22 | 1.54 |
| 7857 | SCG2 | secretogranin II | 2.19 | 1.64 |
| 55777 | MBD5 | methyl-CpG binding domain protein 5 | 2.16 | 1.72 |
| 5420 | PODXL | podocalyxin-like | 2.16 | 1.64 |
| 81627 | TRMT1L | TRM1 tRNA methyltransferase 1-like | 2.13 | 1.62 |
| 22807 | IKZF2 | IKAROS family zinc finger 2 (Helios) | 2.11 | 1.57 |
| 81542 | TMX1 | thioredoxin-related transmembrane protein 1 | 2.08 | 2.07 |
| 10208 | USPL1 | ubiquitin specific peptidase like 1 | 2.08 | 1.66 |
| 151887 | CCDC80 | coiled-coil domain containing 80 | 2.07 | 1.72 |
| 55885 | LMO3 | LIM domain only 3 (rhombotin-like 2) | 2.07 | 1.72 |
| 723961 | INS-IGF2 | INS-IGF2 readthrough | 0.41 | 0.64 |
| 3481 | IGF2 | insulin-like growth factor 2 (somatomedin A) | 0.42 | 0.64 |

Table S6. Transcriptome analysis of HEK293T cells stably overexpressing PLZF (ZBTB16) (Ma(PLZF; H)). The 10 genes with the highest and the 10 genes with the lowest fold changes (relative to the insertless control vector) are shown. Fold changes > 1 (< 1) indicate increased (decreased) mRNA levels. Hypothetical and functionally uncharacterized genes are not given. PLZF and target genes previously published are highlighted in bold and italics, respectively.

| **gene ID** | **gene symbol** | **description** | **fold change Ma(PLZF; H)** |
| --- | --- | --- | --- |
| **7704** | **ZBTB16** | **zinc finger and BTB domain containing 16** | **21.11** |
| 3037 | HAS2 | hyaluronan synthase 2 | 2.93 |
| 27134 | TJP3 | tight junction protein 3 (zona occludens 3) | 2.79 |
| 55511 | SAGE1 | sarcoma antigen 1 | 2.64 |
| 1081 | CGA | glycoprotein hormones, alpha polypeptide | 2.55 |
| 387914 | SHISA2 | shisa homolog 2 (Xenopus laevis) | 2.46 |
| 140731 | ANKRD60 | ankyrin repeat domain 60 | 2.46 |
| 26266 | SLC13A4 | solute carrier family 13 (sodium/sulfate symporters), member 4 | 2.45 |
| 441521 | CT45A5 | cancer/testis antigen family 45, member A5 | 2.39 |
| 10178 | ODZ1 | odz, odd Oz/ten-m homolog 1(Drosophila) | 2.35 |
| … | … | … | … |
| *2626* | *GATA4* | *GATA binding protein 4* | *2.28 [**[[7]](#endnote-8)]* |
| *3399* | *ID3* | *inhibitor of DNA binding 3, dominant negative helix-loop-helix protein* | *1.80* *[[[8]](#endnote-9)]* |
| 84072 | HORMAD1 | HORMA domain containing 1 | 0.17 |
| 6678 | SPARC | secreted protein, acidic, cysteine-rich (osteonectin) | 0.31 |
| 245972 | ATP6V0D2 | ATPase, H+ transporting, lysosomal 38kDa, V0 subunit d2 | 0.34 |
| 51200 | CPA4 | carboxypeptidase A4 | 0.40 |
| 10512 | SEMA3C | sema domain, immunoglobulin domain (Ig), short basic domain, secreted, (semaphorin) 3C | 0.40 |
| 2556 | GABRA3 | gamma-aminobutyric acid (GABA) A receptor, alpha 3 | 0.41 |
| 9512 | PMPCB | peptidase (mitochondrial processing) beta | 0.41 |
| 170690 | ADAMTS16 | ADAM metallopeptidase with thrombospondin type 1 motif, 16 | 0.43 |
| 4325 | MMP16 | matrix metallopeptidase 16 (membrane-inserted) | 0.43 |
| 3491 | CYR61 | cysteine-rich, angiogenic inducer, 61 | 0.44 |

Table S7. Transcriptome analysis of KELLY cells stably overexpressing PLZF (ZBTB16) (Ma(PLZF; K)). The 10 genes with the highest and the 10 genes with the lowest fold changes (relative to the insertless control vector) are shown. Fold changes > 1 (< 1) indicate increased (decreased) mRNA levels. Hypothetical and functionally uncharacterized genes are not given. Target genes previously published and PLZF (ZBTB16) are highlighted in italics and bold, respectively.

| **gene ID** | **gene symbol** | **description** | **fold change Ma(PLZF; K)** |
| --- | --- | --- | --- |
| **7704** | **ZBTB16** | **zinc finger and BTB domain containing 16** | **56.49** |
| 29944 | PNMA3 | paraneoplastic antigen MA3 | 4.79 |
| 2901 | GRIK5 | glutamate receptor, ionotropic, kainate 5 | 4.14 |
| 347732 | PCBD2 | pterin-4 alpha-carbinolamine dehydratase/dimerization cofactor of hepatocyte nuclear factor 1 alpha (TCF1) 2 | 4.03 |
| 9518 | GDF15 | growth differentiation factor 15 | 3.97 |
| 23 | ABCF1 | ATP-binding cassette, sub-family F (GCN20), member 1 | 3.92 |
| 196410 | METTL7B | methyltransferase like 7B | 3.89 |
| 730 | C7 | complement component 7 | 3.78 |
| 29934 | SNX12 | sorting nexin 12 | 3.76 |
| 5737 | PTGFR | prostaglandin F receptor (FP) | 3,71 |
| … | … | … |  |
| *2626* | *GATA4* | *GATA binding protein 4* | *2.39 [Error: Reference source not found]* |
| 6781 | STC1 | stanniocalcin 1 | 0.24 |
| 5272 | SERPINB9 | serpin peptidase inhibitor, clade B (ovalbumin), member 9 | 0.29 |
| 4782 | NFIC | nuclear factor I/C (CCAAT-binding transcription factor) | 0.30 |
| 8793 | TNFRSF10D | tumor necrosis factor receptor superfamily, member 10d, decoy with truncated death domain | 0.31 |
| 4281 | MID1 | midline 1 (Opitz/BBB syndrome) | 0.32 |
| 23294 | ANKS1A | ankyrin repeat and sterile alpha motif domain containing 1A | 0.32 |
| 124274 | GPR139 | G protein-coupled receptor 139 | 0.33 |
| 91947 | ARRDC4 | arrestin domain containing 4 | 0.34 |
| 1392 | CRH | corticotropin releasing hormone | 0.35 |
| 80256 | KIAA1539 | KIAA1539 | 0.35 |
| … | … | … | … |
| *3397* | *ID1* | *inhibitor of DNA binding 1, dominant negative helix-loop-helix protein* | *0.40 [Error: Reference source not found]* |
| *3398* | *ID2* | *inhibitor of DNA binding 2B, dominant negative helix-loop-helix protein (pseudogene) /// inhibitor of DNA binding 2, dominant negative helix-loop-helix protein* | *0.44 [[[9]](#endnote-10)]* |
| *3399* | *ID3* | *inhibitor of DNA binding 3, dominant negative helix-loop-helix protein* | *0.50 [Error: Reference source not found]* |

Table S8. Transcriptome analysis of KELLY cells after incubation with genistein (Ma(geni)). The 10 genes with the highest and the 10 genes with the lowest fold changes (relative to a DMSO control) are shown. Fold changes > 1 (< 1) indicate increased (decreased) mRNA levels. Hypothetical and functionally uncharacterized genes are not given.

| **gene ID** | **gene symbol** | **description** | **fold change Ma(geni)** |
| --- | --- | --- | --- |
| 677827 | SNORA46 | small nucleolar RNA, H/ACA box 46 | 6.28 |
| 1470 | CST2 | cystatin SA | 4.76 |
| 677801 | SNORA14A | small nucleolar RNA, H/ACA box 14A | 3.36 |
| 677802 | SNORA14B | small nucleolar RNA, H/ACA box 14B | 3.36 |
| 4069 | LYZ | lysozyme | 3.18 |
| 6414 | SEPP1 | selenoprotein P, plasma, 1 | 2.69 |
| 677811 | SNORA28 | small nucleolar RNA, H/ACA box 28 | 2.58 |
| 7805 | LAPTM5 | lysosomal protein transmembrane 5 | 2.51 |
| 5341 | PLEK | pleckstrin | 2.50 |
| 654321 | SNORA75 | small nucleolar RNA, H/ACA box 75 | 2.41 |
| 4642 | MYO1D | myosin ID | 0.44 |
| 7813 | EVI5 | ecotropic viral integration site 5 | 0.46 |
| 3696 | ITGB8 | integrin, beta 8 | 0.53 |
| 114987 | WDR31 | WD repeat domain 31 | 0.53 |
| 54454 | ATAD2B | ATPase family, AAA domain containing 2B | 0.54 |
| 113802 | HENMT1 | HEN1 methyltransferase homolog 1 (Arabidopsis) | 0.54 |
| 153733 | CCDC112 | coiled-coil domain containing 112 | 0.55 |
| 100526761 | C13orf38-SOHLH2 | C13orf38-SOHLH2 readthrough | 0.55 |
| 79807 | GSTCD | glutathione S-transferase, C-terminal domain containing | 0.55 |

Table S9. Transcripts with the highest fold changes within the intersection of Ma(geni), Ma(PLZF; K, H). Fold changes > 1 indicate increased mRNA levels. Hypothetical and functionally uncharacterized genes are not given.

| **gene ID** | **gene symbol** | **description** | **fold change Ma(geni)** | **fold change Ma(PLZF; K)** | **fold change Ma(PLZF; H)** |
| --- | --- | --- | --- | --- | --- |
| 3488 | IGFBP5 | insulin-like growth factor binding protein 5 | 1.59 | 2.16 | 1.99 |
| 3778 | KCNMA1 | potassium large conductance calcium-activated channel, subfamily M, alpha member 1 | 1.61 | 2.03 | 1.82 |
| 4241 | MFI2 | antigen p97 (melanoma associated) identified by monoclonal antibodies 133.2 and 96.5 | 1.58 | 2.36 | 1.68 |
| 5649 | RELN | reelin | 1.57 | 2.10 | 1.79 |
| 6261 | RYR1 | ryanodine receptor 1 (skeletal) | 1.51 | 2.06 | 1.66 |
| 6263 | RYR3 | ryanodine receptor 3 | 1.65 | 2.01 | 1.73 |
| 7706 | TRIM25 | tripartite motif containing 25 | 1.67 | 2.06 | 1.72 |
| 8395 | PIP5K1B | phosphatidylinositol-4-phosphate 5-kinase, type I, beta | 1.59 | 2.16 | 1.69 |
| 8986 | RPS6KA4 | ribosomal protein S6 kinase, 90kDa, polypeptide 4 | 1.61 | 3.01 | 1.62 |
| 9466 | IL27RA | interleukin 27 receptor, alpha | 1.54 | 2.20 | 1.74 |
| 23129 | PLXND1 | plexin D1 | 1.61 | 2.07 | 1.75 |
| 53353 | LRP1B | low density lipoprotein receptor-related protein 1B | 1.61 | 2.20 | 1.80 |
| 55040 | EPN3 | epsin 3 | 1.59 | 2.30 | 1.77 |
| 64399 | HHIP | hedgehog interacting protein | 1.59 | 2.38 | 1.65 |
| 64411 | ARAP3 | ArfGAP with RhoGAP domain, ankyrin repeat and PH domain 3 | 1.55 | 2.14 | 1.67 |
| 79705 | LRRK1 | leucine-rich repeat kinase 1 | 1.61 | 2.06 | 1.62 |
| 81704 | DOCK8 | dedicator of cytokinesis 8 | 1.96 | 2.13 | 1.66 |
| 84467 | FBN3 | fibrillin 3 | 1.65 | 2.27 | 1.78 |
| 93035 | PKHD1L1 | polycystic kidney and hepatic disease 1 (autosomal recessive)-like 1 | 1.84 | 2.06 | 1.64 |
| 147409 | DSG4 | desmoglein 4 | 1.64 | 2.01 | 1.66 |

Table S10. Transcriptome comparison of Ma(PLZF; H, K) and Ma(si(P)RR) results. The table is based on the transcripts with the highest fold changes according to Ma(PLZF; K). Gene IDs, gene symbols and descriptions (derived from Eldorado database) of the respective transcripts are shown. Fold changes > 1 (< 1) indicate increased (decreased) mRNA levels. Hypothetical and functionally uncharacterized genes are not given.

| **gene ID** | **gene symbol** | **description** | **fold change Ma(PLZF; K)** | **fold change Ma(si(P)RR)** |
| --- | --- | --- | --- | --- |
| 2043 | EPHA4 | EPH receptor A4 | 2.38 | 1.75 |
| 1272 | CNTN1 | contactin 1 | 2.35 | 2.20 |
| 1803 | DPP4 | dipeptidyl-peptidase 4 | 2.33 | 1.80 |
| 144568 | A2ML1 | alpha-2-macroglobulin-like 1 | 2.31 | 1.87 |
| 3910 | LAMA4 | laminin, alpha 4 | 2.27 | 1.87 |
| 168667 | BMPER | BMP binding endothelial regulator | 2.27 | 1.68 |
| 22941 | SHANK2 | SH3 and multiple ankyrin repeat domains 2 | 2.27 | 1.92 |
| 8997 | KALRN | kalirin, RhoGEF kinase | 2.23 | 1.69 |
| 84033 | OBSCN | Obscuring, cytoskeletal calmodulin and titin-interacting RhoGEF | 2.22 | 1.82 |
| 148398 | SAMD11 | sterile alpha motif domain containing 11 | 2.20 | 1.82 |

Table S11. Intersection of ChIP-chip and microarray datasets. ChIP-chip analyses revealed 222 genes on which pol II recruitment was enriched by PLZF. This dataset was intersected with microarray Ma(PLZF; K) resulting in 66 genes whose mRNA level was increased. The top ten with the highest fold changes are given.

| **gene ID** | **gene symbol** | **description** | **fold change Ma(PLZF; K)** |
| --- | --- | --- | --- |
| 6373 | CXCL11 | chemokine (C-X-C motif) ligand 11 | 3.58 |
| 25913 | POT1 | protection of telomeres 1 homolog (S. pombe) | 3.10 |
| 5356 | PLRG1 | pleiotropic regulator 1 (PRL1 homolog, Arabidopsis) | 2.79 |
| 9604 | RNF14 | ring finger protein 14 | 2.64 |
| 8637 | EIF4EBP3 | eukaryotic translation initiation factor 4E binding protein 3 | 2.64 |
| 22872 | SEC31A | SEC31 homolog A (S. cerevisiae) | 2.62 |
| 55133 | SRBD1 | S1 RNA binding domain 1 | 2.55 |
| 2554 | GABRA1 | gamma-aminobutyric acid (GABA) A receptor, alpha 1 | 2.53 |
| 4594 | MUT | methylmalonyl CoA mutase | 2.53 |
| 10827 | FAM114A2 | family with sequence similarity 114, member A2 | 2.53 |

Table S12. Specific genes constituting the transcript clusters associated with brain and forebrain development shown in table 2. The functional summary is based on the Eldorado database (version E24R1103; Genomatix). 1: Ma(si(P)RR), 2: Ma(PLZF; H, K)/ brain development, 3: Ma(PLZF; H, K)/ forebrain development.

| **gene symbol (gene ID)** | **description: functional summary** | **1** | **2** | **3** |
| --- | --- | --- | --- | --- |
| ADAM19 (8728) | ADAM metallopeptidase domain 19: ADAM metallopeptidase domain 19: This gene encodes a member of the ADAM (a disintegrin and metalloprotease domain) family. Members of this family are membrane-anchored proteins structurally related to snake venom disintegrins, and have been implicated in a variety of biological processes involving cell-cell and cell-matrix interactions, including fertilization, muscle development, and neurogenesis. This member is a type I transmembrane protein and serves as a marker for dendritic cell differentiation. It has also been demonstrated to be an active metalloproteinase, which may be involved in normal physiological and pathological processes such as cells migration, cell adhesion, cell-cell and cell-matrix interactions, and signal transduction. [provided by RefSeq] | + | - | - |
| AHI1  (54806) | Abelson helper integration site 1: Abelson helper integration site 1: This gene is apparently required for both cerebellar and cortical development in humans. This gene mutations cause specific forms of Joubert syndrome-related disorders. Joubert syndrome (JS) is a recessively inherited developmental brain disorder with several identified causative chromosomal loci. Alternatively spliced transcript variants encoding different isoforms have been identified. [provided by RefSeq] | + | - | - |
| ATM  (472) | ataxia telangiectasia mutated: The protein encoded by this gene belongs to the PI3/PI4-kinase family. This protein is an important cell cycle checkpoint kinase that phosphorylates; thus, it functions as a regulator of a wide variety of downstream proteins, including tumor suppressor proteins p53 and BRCA1, checkpoint kinase CHK2, checkpoint proteins RAD17 and RAD9, and DNA repair protein NBS1. This protein and the closely related kinase ATR are thought to be master controllers of cell cycle checkpoint signaling pathways that are required for cell response to DNA damage and for genome stability. Mutations in this gene are associated with ataxia telangiectasia, an autosomal recessive disorder. [provided by RefSeq] | + | - | - |
| ATP2B2  (491) | ATPase, Ca++ transporting, plasma membrane 2: The protein encoded by this gene belongs to the family of P-type primary ion transport ATPases characterized by the formation of an aspartyl phosphate intermediate during the reaction cycle. These enzymes remove bivalent calcium ions from eukaryotic cells against very large concentration gradients and play a critical role in intracellular calcium homeostasis. The mammalian plasma membrane calcium ATPase isoforms are encoded by at least four separate genes and the diversity of these enzymes is further increased by alternative splicing of transcripts. The expression of different isoforms and splice variants is regulated in a developmental, tissue- and cell type-specific manner, suggesting that these pumps are functionally adapted to the physiological needs of particular cells and tissues. This gene encodes the plasma membrane calcium ATPase isoform 2. Alternatively spliced transcript variants encoding different isoforms have been identified. [provided by RefSeq] | + | - | - |
| ATRX  (546) | alpha thalassemia/mental retardation syndrome X-linked: The protein encoded by this gene contains an ATPase/helicase domain, and thus it belongs to the SWI/SNF family of chromatin remodeling proteins. The mutations of this gene are associated with an X-linked mental retardation (XLMR) syndrome most often accompanied by alpha-thalassemia (ATRX) syndrome. These mutations have been shown to cause diverse changes in the pattern of DNA methylation, which may provide a link between chromatin remodeling, DNA methylation, and gene expression in developmental processes. This protein is found to undergo cell cycle-dependent phosphorylation, which regulates its nuclear matrix and chromatin association, and suggests its involvement in the gene regulation at interphase and chromosomal segregation in mitosis. Multiple alternatively spliced transcript variants encoding distinct isoforms have been reported. [provided by RefSeq] | + | - | - |
| BCL2  (596) | B-cell CLL/lymphoma 2: This gene encodes an integral outer mitochondrial membrane protein that blocks the apoptotic death of some cells such as lymphocytes. Constitutive expression of BCL2, such as in the case of translocation of BCL2 to Ig heavy chain locus, is thought to be the cause of follicular lymphoma. Two transcript variants, produced by alternate splicing, differ in their C-terminal ends. [provided by RefSeq] | + | - | - |
| BRCA2  (675) | breast cancer 2, early onset: Inherited mutations in BRCA1 and this gene, BRCA2, confer increased lifetime risk of developing breast or ovarian cancer. Both BRCA1 and BRCA2 are involved in maintenance of genome stability, specifically the homologous recombination pathway for double-strand DNA repair. The BRCA2 protein contains several copies of a 70 aa motif called the BRC motif, and these motifs mediate binding to the RAD51 recombinase which functions in DNA repair. BRCA2 is considered a tumor suppressor gene, as tumors with BRCA2 mutations generally exhibit loss of heterozygosity (LOH) of the wild-type allele. [provided by RefSeq] | + | - | - |
| CACNA1A (773) | calcium channel, voltage-dependent, P/Q type, alpha 1A subunit: Voltage-dependent calcium channels mediate the entry of calcium ions into excitable cells, and are also involved in a variety of calcium-dependent processes, including muscle contraction, hormone or neurotransmitter release, and gene expression. Calcium channels are multisubunit complexes composed of alpha-1, beta, alpha-2/delta, and gamma subunits. The channel activity is directed by the pore-forming alpha-1 subunit, whereas, the others act as auxiliary subunits regulating this activity. The distinctive properties of the calcium channel types are related primarily to the expression of a variety of alpha-1 isoforms, alpha-1A, B, C, D, E, and S. This gene encodes the alpha-1A subunit, which is predominantly expressed in neuronal tissue. Mutations in this gene are associated with 2 neurologic disorders, familial hemiplegic migraine and episodic ataxia 2. This gene also exhibits polymorphic variation due to (CAG)n-repeats. Multiple transcript variants encoding different isoforms have been found for this gene. In one set of transcript variants, the (CAG)n-repeats occur in the 3&apos; UTR, and are not associated with any disease. But in another set of variants, an insertion extends the coding region to include the (CAG)n-repeats which encode a polyglutamine tract. Expansion of the (CAG)n-repeats from the normal 4-16 to 21-28 in the coding region is associated with spinocerebellar ataxia 6. [provided by RefSeq] | + | - | - |
| CEP120 (153241) | centrosomal protein 120kDa: This gene encodes a protein that functions in the microtubule-dependent coupling of the nucleus and the centrosome. A similar protein in mouse plays a role in both interkinetic nuclear migration, which is a characteristic pattern of nuclear movement in neural progenitors, and in neural progenitor self-renewal. Mutations in this gene are predicted to result in neurogenic defects. Alternative splicing results in multiple transcript variants. [provided by RefSeq] | + | - | - |
| CEP290 (80184) | centrosomal protein 290kDa: This gene encodes a protein with 13 putative coiled-coil domains, a region with homology to SMC chromosome segregation ATPases, six KID motifs, three tropomyosin homology domains and an ATP/GTP binding site motif A. The protein is localized to the centrosome and cilia and has sites for N-glycosylation, tyrosine sulfation, phosphorylation, N-myristoylation, and amidation. Mutations in this gene have been associated with Joubert syndrome and nephronophthisis and the presence of antibodies against this protein is associated with several forms of cancer. [provided by RefSeq] | + | - | - |
| CNTN1  (1272) | contactin 1: The protein encoded by this gene is a member of the immunoglobulin superfamily. It is a glycosylphosphatidylinositol (GPI)-anchored neuronal membrane protein that functions as a cell adhesion molecule. It may play a role in the formation of axon connections in the developing nervous system. Two alternatively spliced transcript variants encoding different isoforms have been described for this gene. [provided by RefSeq] | + | + | - |
| CREB1  (1385) | cAMP responsive element binding protein 1: This gene encodes a transcription factor that is a member of the leucine zipper family of DNA binding proteins. This protein binds as a homodimer to the cAMP-responsive element, an octameric palindrome. The protein is phosphorylated by several protein kinases, and induces transcription of genes in response to hormonal stimulation of the cAMP pathway. Alternate splicing of this gene results in two transcript variants encoding different isoforms. [provided by RefSeq] | + | - | - |
| DCX  (1641) | doublecortin: This gene encodes a member of the doublecortin family. The protein encoded by this gene is a cytoplasmic protein and contains two doublecortin domains, which bind microtubules. In the developing cortex, cortical neurons must migrate over long distances to reach the site of their final differentiation. The encoded protein appears to direct neuronal migration by regulating the organization and stability of microtubules. In addition, the encoded protein interacts with LIS1, the regulatory gamma subunit of platelet activating factor acetylhydrolase, and this interaction is important to proper microtubule function in the developing cortex. Mutations in this gene cause abnormal migration of neurons during development and disrupt the layering of the cortex, leading to epilepsy, mental retardation, subcortical band heterotopia (&quot;double cortex&quot; syndrome) in females and lissencephaly (&quot;smooth brain&quot; syndrome) in males. Multiple transcript variants encoding different isoforms have been found for this gene. [provided by RefSeq] | + | - | - |
| DKK1 (22943) | dickkopf homolog 1 (Xenopus laevis): This gene encodes a protein that is a member of the dickkopf family. It is a secreted protein with two cysteine rich regions and is involved in embryonic development through its inhibition of the WNT signaling pathway. Elevated levels of DKK1 in bone marrow plasma and peripheral blood is associated with the presence of osteolytic bone lesions in patients with multiple myeloma. [provided by RefSeq] | + | - | - |
| DYNC2H1 (79659) | dynein, cytoplasmic 2, heavy chain 1: This gene encodes a large cytoplasmic dynein protein that is involved in retrograde transport in the cilium and has a role in intraflagellar transport, a process required for ciliary/flagellar assembly. Mutations in this gene cause a heterogeneous spectrum of conditions related to altered primary cilium function and often involve polydactyly, abnormal skeletogenesis, and polycystic kidneys. Alternative splicing results in multiple transcript variants encoding distinct proteins. [provided by RefSeq] | + | - | - |
| EGR1  (1958) | early growth response 1: The protein encoded by this gene belongs to the EGR family of C2H2-type zinc-finger proteins. It is a nuclear protein and functions as a transcriptional regulator. The products of target genes it activates are required for differentitation and mitogenesis. Studies suggest this is a cancer suppresor gene. [provided by RefSeq] | + | - | - |
| EOMES (8320) | eomesodermin: This gene encodes a member of a conserved protein family that shares a common DNA-binding domain, the T-box. T-box genes encode transcription factors involved in the regulation of developmental processes. A similiar gene disrupted in mice is shown to be essential during trophoblast development and gastrulation. [provided by RefSeq] | + | - | - |
| EPHA4 (2043) | EPH receptor A4: This gene belongs to the ephrin receptor subfamily of the protein-tyrosine kinase family. EPH and EPH-related receptors have been implicated in mediating developmental events, particularly in the nervous system. Receptors in the EPH subfamily typically have a single kinase domain and an extracellular region containing a Cys-rich domain and 2 fibronectin type III repeats. The ephrin receptors are divided into 2 groups based on the similarity of their extracellular domain sequences and their affinities for binding ephrin-A and ephrin-B ligands. [provided by RefSeq] | + | + | + |
| EPHA7 (2045) | EPH receptor A7: This gene belongs to the ephrin receptor subfamily of the protein-tyrosine kinase family. EPH and EPH-related receptors have been implicated in mediating developmental events, particularly in the nervous system. Receptors in the EPH subfamily typically have a single kinase domain and an extracellular region containing a Cys-rich domain and 2 fibronectin type III repeats. The ephrin receptors are divided into 2 groups based on the similarity of their extracellular domain sequences and their affinities for binding ephrin-A and ephrin-B ligands. [provided by RefSeq] | + | - | - |
| FOXC1 (2296) | forkhead box C1: This gene belongs to the forkhead family of transcription factors which is characterized by a distinct DNA-binding forkhead domain. The specific function of this gene has not yet been determined; however, it has been shown to play a role in the regulation of embryonic and ocular development. Mutations in this gene cause various glaucoma phenotypes including primary congenital glaucoma, autosomal dominant iridogoniodysgenesis anomaly, and Axenfeld-Rieger anomaly. [provided by RefSeq] | + | - | - |
| GNPAT (8443) | glyceronephosphate O-acyltransferase: This gene encodes an enzyme located in the peroxisomal membrane which is essential to the synthesis of ether phospholipids. Mutations in this gene are associated with rhizomelic chondrodysplasia punctata. [provided by RefSeq] | + | - | - |
| HES1  (3280) | hairy and enhancer of split 1, (Drosophila): This protein belongs to the basic helix-loop-helix family of transcription factors. It is a transcriptional repressor of genes that require a bHLH protein for their transcription. The protein has a particular type of basic domain that contains a helix interrupting protein that binds to the N-box rather than the canonical E-box. [provided by RefSeq] | + | - | - |
| HESX1 (8820) | HESX homeobox 1: This gene encodes a conserved homeobox protein that is a transcriptional repressor in the developing forebrain and pituitary gland. Mutations in this gene are associated with septooptic dysplasia, HESX1-related growth hormone deficiency, and combined pituitary hormone deficiency. [provided by RefSeq] | + | - | - |
| ID4  (3400) | inhibitor of DNA binding 4, dominant negative helix-loop-helix protein: Transcription factors containing a basic helix-loop-helix (bHLH) motif regulate expression of tissue-specific genes in a number of mammalian and insect systems. DNA-binding activity of the bHLH proteins is dependent on formation of homo- and/or heterodimers. Dominant-negative HLH proteins encoded by Id-related genes, such as ID4, also contain the HLH-dimerization domain but lack the DNA-binding basic domain. Consequently, Id proteins inhibit binding to DNA and transcriptional transactivation by heterodimerization with bHLH proteins (Pagliuca et al., 1995 [PubMed 7665172]).[supplied by OMIM] | + | - | - |
| IFT88  (8100) | intraflagellar transport 88 homolog (Chlamydomonas): This gene encodes a member of the tetratrico peptide repeat (TPR) family. Mutations of a similar gene in mouse can cause polycystic kidney disease. Two transcript variants encoding distinct isoforms have been identified for this gene. [provided by RefSeq] | + | - | - |
| IRS2  (8660) | insulin receptor substrate 2: This gene encodes the insulin receptor substrate 2, a cytoplasmic signaling molecule that mediates effects of insulin, insulin-like growth factor 1, and other cytokines by acting as a molecular adaptor between diverse receptor tyrosine kinases and downstream effectors. The product of this gene is phosphorylated by the insulin receptor tyrosine kinase upon receptor stimulation, as well as by an interleukin 4 receptor-associated kinase in response to IL4 treatment. [provided by RefSeq] | + | - | - |
| LEF1 (51176) | lymphoid enhancer-binding factor 1: This gene encodes a transcription factor belonging to a family of proteins that share homology with the high mobility group protein-1. The protein encoded by this gene can bind to a functionally important site in the T-cell receptor-alpha enhancer, thereby conferring maximal enhancer activity. This transcription factor is involved in the Wnt signaling pathway, and it may function in hair cell differentiation and follicle morphogenesis. Mutations in this gene have been found in somatic sebaceous tumors. This gene has also been linked to other cancers, including androgen-independent prostate cancer. Alternative splicing results in multiple transcript variants. [provided by RefSeq] | + | - | - |
| LRP2  (4036) | low density lipoprotein receptor-related protein 2: The protein encoded by this gene, low density lipoprotein-related protein 2 (LRP2) or megalin, is a multi-ligand endocytic receptor that is expressed in many different tissues but primarily in absorptive epithilial tissues such as the kidney. This glycoprotein has a large amino-terminal extracellular domain, a single transmembrane domain, and a short carboxy-terminal cytoplasmic tail. The extracellular ligand-binding-domains bind diverse macromolecules including albumin, apolipoproteins B and E, and lipoprotein lipase. The LRP2 protein is critical for the reuptake of numerous ligands, including lipoproteins, sterols, vitamin-binding proteins, and hormones. This protein also has a role in cell-signaling; extracellular ligands include parathyroid horomones and the morphogen sonic hedgehog while cytosolic ligands include MAP kinase scaffold proteins and JNK interacting proteins. Recycling of this membrane receptor is regulated by phosphorylation of its cytoplasmic domain. Mutations in this gene cause Donnai-Barrow syndrome (DBS) and facio-oculoacoustico-renal syndrome (FOAR). | + | + | + |
| MAPK8 (5599) | mitogen-activated protein kinase 8: The protein encoded by this gene is a member of the MAP kinase family. MAP kinases act as an integration point for multiple biochemical signals, and are involved in a wide variety of cellular processes such as proliferation, differentiation, transcription regulation and development. This kinase is activated by various cell stimuli, and targets specific transcription factors, and thus mediates immediate-early gene expression in response to cell stimuli. The activation of this kinase by tumor-necrosis factor alpha (TNF-alpha) is found to be required for TNF-alpha induced apoptosis. This kinase is also involved in UV radiation induced apoptosis, which is thought to be related to cytochrom c-mediated cell death pathway. Studies of the mouse counterpart of this gene suggested that this kinase play a key role in T cell proliferation, apoptosis and differentiation. Four alternatively spliced transcript variants encoding distinct isoforms have been reported. [provided by RefSeq] | + | - | - |
| MAPK9 (5601) | mitogen-activated protein kinase 9: The protein encoded by this gene is a member of the MAP kinase family. MAP kinases act as an integration point for multiple biochemical signals, and are involved in a wide variety of cellular processes such as proliferation, differentiation, transcription regulation and development. This kinase targets specific transcription factors, and thus mediates immediate-early gene expression in response to various cell stimuli. It is most closely related to MAPK8, both of which are involved in UV radiation induced apoptosis, thought to be related to the cytochrome c-mediated cell death pathway. This gene and MAPK8 are also known as c-Jun N-terminal kinases. This kinase blocks the ubiquitination of tumor suppressor p53, and thus it increases the stability of p53 in nonstressed cells. Studies of this gene&apos;s mouse counterpart suggest a key role in T-cell differentiation. Several alternatively spliced transcript variants encoding distinct isoforms have been reported. [provided by RefSeq] | + | - | - |
| MARCKS (4082) | myristoylated alanine-rich protein kinase C substrate: The protein encoded by this gene is a substrate for protein kinase C. It is localized to the plasma membrane and is an actin filament crosslinking protein. Phosphorylation by protein kinase C or binding to calcium-calmodulin inhibits its association with actin and with the plasma membrane, leading to its presence in the cytoplasm. The protein is thought to be involved in cell motility, phagocytosis, membrane trafficking and mitogenesis. [provided by RefSeq] | + | - | - |
| MDM2 (4193) | Mdm2 p53 binding protein homolog (mouse): This gene is a target gene of the transcription factor tumor protein p53. The encoded protein is a nuclear phosphoprotein that binds and inhibits transactivation by tumor protein p53, as part of an autoregulatory negative feedback loop. Overexpression of this gene can result in excessive inactivation of tumor protein p53, diminishing its tumor suppressor function. This protein has E3 ubiquitin ligase activity, which targets tumor protein p53 for proteasomal degradation. This protein also affects the cell cycle, apoptosis, and tumorigenesis through interactions with other proteins, including retinoblastoma 1 and ribosomal protein L5. More than 40 different alternatively spliced transcript variants have been isolated from both tumor and normal tissues. [provided by RefSeq] | + | - | - |
| MECP2 (4204) | methyl CpG binding protein 2 (Rett syndrome): DNA methylation is the major modification of eukaryotic genomes and plays an essential role in mammalian development. Human proteins MECP2, MBD1, MBD2, MBD3, and MBD4 comprise a family of nuclear proteins related by the presence in each of a methyl-CpG binding domain (MBD). Each of these proteins, with the exception of MBD3, is capable of binding specifically to methylated DNA. MECP2, MBD1 and MBD2 can also repress transcription from methylated gene promoters. In contrast to other MBD family members, MECP2 is X-linked and subject to X inactivation. MECP2 is dispensible in stem cells, but is essential for embryonic development. MECP2 gene mutations are the cause of most cases of Rett syndrome, a progressive neurologic developmental disorder and one of the most common causes of mental retardation in females. [provided by RefSeq] | + | - | - |
| NCAM1 (4684) | neural cell adhesion molecule 1: No summary found | + | - | - |
| NF1  (4763) | neurofibromin 1: This gene product appears to function as a negative regulator of the ras signal transduction pathway. Mutations in this gene have been linked to neurofibromatosis type 1, juvenile myelomonocytic leukemia and Watson syndrome. The mRNA for this gene is subject to RNA editing (CGA&gt;UGA-&gt;Arg1306Term) resulting in premature translation termination. Alternatively spliced transcript variants encoding different isoforms have also been described for this gene. [provided by RefSeq] | + | - | - |
| NIPBL (25836) | Nipped-B homolog (Drosophila): This gene encodes the homolog of the Drosophila melanogaster Nipped-B gene product and fungal Scc2-type sister chromatid cohesion proteins. The Drosophila protein facilitates enhancer-promoter communication of remote enhancers and plays a role in developmental regulation. It is also homologous to a family of chromosomal adherins with broad roles in sister chromatid cohesion, chromosome condensation, and DNA repair. The human protein has a bipartite nuclear targeting sequence and a putative HEAT repeat. Condensins, cohesins and other complexes with chromosome-related functions also contain HEAT repeats. Mutations in this gene result in Cornelia de Lange syndrome, a disorder characterized by dysmorphic facial features, growth delay, limb reduction defects, and mental retardation. Two transcript variants encoding different isoforms have been found for this gene. [provided by RefSeq] | + | - | - |
| NLGN4X (57502) | neuroligin 4, X-linked: This gene encodes a member of a family of neuronal cell surface proteins. Members of this family may act as splice site-specific ligands for beta-neurexins and may be involved in the formation and remodeling of central nervous system synapses. The encoded protein interacts with discs, large (Drosophila) homolog 4 (DLG4). Mutations in this gene have been associated with autism and Asperger syndrome. Two transcript variants encoding the same protein have been identified for this gene. [provided by RefSeq] | + | - | - |
| NR4A3 (8013) | nuclear receptor subfamily 4, group A, member 3: This gene encodes a member of the steroid-thyroid hormone-retinoid receptor superfamily. The encoded protein may act as a transcriptional activator. The protein can efficiently bind the NGFI-B Response Element (NBRE). Three different versions of extraskeletal myxoid chondrosarcomas (EMCs) are the result of reciprocal translocations between this gene and other genes. The translocation breakpoints are associated with Nuclear Receptor Subfamily 4, Group A, Member 3 (on chromosome 9) and either Ewing Sarcome Breakpoint Region 1 (on chromosome 22), RNA Polymerase II, TATA Box-Binding Protein-Associated Factor, 68-KD (on chromosome 17), or Transcription factor 12 (on chromosome 15). Multiple transcript variants encoding different isoforms have been found for this gene. [provided by RefSeq] | + | - | - |
| PLXNA4 (91584) | plexin A4: No summary found | + | - | - |
| PTEN  (5728) | phosphatase and tensin homolog: This gene was identified as a tumor suppressor that is mutated in a large number of cancers at high frequency. The protein encoded this gene is a phosphatidylinositol-3,4,5-trisphosphate 3-phosphatase. It contains a tensin like domain as well as a catalytic domain similar to that of the dual specificity protein tyrosine phosphatases. Unlike most of the protein tyrosine phosphatases, this protein preferentially dephosphorylates phosphoinositide substrates. It negatively regulates intracellular levels of phosphatidylinositol-3,4,5-trisphosphate in cells and functions as a tumor suppressor by negatively regulating AKT/PKB signaling pathway. [provided by RefSeq] | + | - | - |
| SEPP1 (6414) | selenoprotein P, plasma, 1: This gene encodes a selenoprotein containing multiple selenocysteine (Sec) residues, which are encoded by the UGA codon that normally signals translation termination. The 3&apos; UTR of selenoprotein genes have a common stem-loop structure, the sec insertion sequence (SECIS), which is necessary for the recognition of UGA as a Sec codon rather than as a stop signal. This selenoprotein is an extracellular glycoprotein, and is unusual in that it contains 10 Sec residues per polypeptide. It is a heparin-binding protein that appears to be associated with endothelial cells, and has been implicated to function as an antioxidant in the extracellular space. Several transcript variants, encoding either the same or different isoform, have been found for this gene. [provided by RefSeq] | + | - | - |
| SLIT2  (9353) | slit homolog 2 (Drosophila): No summary found | + | - | - |
| SMARCA1 (6594) | SWI/SNF related, matrix associated, actin dependent regulator of chromatin, subfamily a, member 1: This gene encodes a member of the SWI/SNF family of proteins. Members of this family have helicase and ATPase activities and are thought to regulate transcription of certain genes by altering the chromatin structure around those genes. Alternatively spliced transcript variants encoding different isoforms have been found for this gene. [provided by RefSeq] | + | - | - |
| SOX4  (6659) | SRY (sex determining region Y)-box 4: This intronless gene encodes a member of the SOX (SRY-related HMG-box) family of transcription factors involved in the regulation of embryonic development and in the determination of the cell fate. The encoded protein may act as a transcriptional regulator after forming a protein complex with other proteins, such as syndecan binding protein (syntenin). The protein may function in the apoptosis pathway leading to cell death as well as to tumorigenesis and may mediate downstream effects of parathyroid hormone (PTH) and PTH-related protein (PTHrP) in bone development. The solution structure has been resolved for the HMG-box of a similar mouse protein. [provided by RefSeq] | + | - | - |
| STXBP3 (6814) | syntaxin binding protein 3: No summary found | + | - | - |
| THBS2 (7058) | thrombospondin 2: The protein encoded by this gene belongs to the thrombospondin family. It is a disulfide-linked homotrimeric glycoprotein that mediates cell-to-cell and cell-to-matrix interactions. This protein has been shown to function as a potent inhibitor of tumor growth and angiogenesis. Studies of the mouse counterpart suggest that this protein may modulate the cell surface properties of mesenchymal cells and be involved in cell adhesion and migration. [provided by RefSeq] | + | - | - |
| ZEB2  (9839) | zinc finger E-box binding homeobox 2: The protein encoded by this gene is a member of the Zfh1 family of 2-handed zinc finger/homeodomain proteins. It is located in the nucleus and functions as a DNA-binding transcriptional repressor that interacts with activated SMADs. Mutations in this gene are associated with Hirschsprung disease/Mowat-Wilson syndrome. Alternatively spliced transcript variants have been found for this gene. | + | - | - |
| ZIC5  (85416) | Zic family member 5 (odd-paired homolog, Drosophila): This gene encodes a member of the ZIC family of C2H2-type zinc finger proteins. Members of this family are important during development, and have been associated X-linked visceral heterotaxy and holoprosencephaly type 5. This gene is closely linked to a gene encoding zinc finger protein of the cerebellum 2, a related family member on chromosome 13. [provided by RefSeq] | + | - | - |
| ZNF423 (23090) | zinc finger protein 423: The protein encoded by this gene is a nuclear protein that belongs to the family of Kruppel-like C2H2 zinc finger proteins. It functions as a DNA-binding transcription factor by using distinct zinc fingers in different signaling pathways. Thus, it is thought that this gene may have multiple roles in signal transduction during development. [provided by RefSeq] | + | - | - |
| ADCYAP1R1 (117) | adenylate cyclase activating polypeptide 1 (pituitary) receptor type I: This gene encodes type I adenylate cyclase activating polypeptide receptor, which is a membrane-associated protein and shares significant homology with members of the glucagon/secretin receptor family. This receptor mediates diverse biological actions of adenylate cyclase activating polypeptide 1 and is positively coupled to adenylate cyclase. Multiple alternatively spliced transcript variants encoding distinct isoforms have been identified. [provided by RefSeq] | - | + | + |
| ALDH1A2 (8854) | aldehyde dehydrogenase 1 family, member A2: This protein belongs to the aldehyde dehydrogenase family of proteins. The product of this gene is an enzyme that catalyzes the synthesis of retinoic acid (RA) from retinaldehyde. Retinoic acid, the active derivative of vitamin A (retinol), is a hormonal signaling molecule that functions in developing and adult tissues. The studies of a similar mouse gene suggest that this enzyme and the cytochrome CYP26A1, concurrently establish local embryonic retinoic acid levels which facilitate posterior organ development and prevent spina bifida. Three transcript variants encoding distinct isoforms have been identified for this gene. [provided by RefSeq] | - | + | + |
| ALDH1A3 (220) | aldehyde dehydrogenase 1 family, member A3: Aldehyde dehydrogenase isozymes are thought to play a major role in the detoxification of aldehydes generated by alcohol metabolism and lipid peroxidation. The enzyme encoded by this gene uses retinal as a substrate, either in a free or cellular retinol-binding protein form. [provided by RefSeq] | - | + | + |
| DLX1  (1745) | distal-less homeobox 1: This gene encodes a member of a homeobox transcription factor gene family similiar to the Drosophila distal-less gene. The encoded protein is localized to the nucleus where it may function as a transcriptional regulator of signals from multiple TGF-{beta} superfamily members. The encoded protein may play a role in the control of craniofacial patterning and the differentiation and survival of inhibitory neurons in the forebrain. This gene is located in a tail-to-tail configuration with another member of the family on the long arm of chromosome 2. Alternatively spliced transcript variants encoding different isoforms have been described. [provided by RefSeq] | - | + | + |
| EPHB2 (2048) | EPH receptor B2: Ephrin receptors and their ligands, the ephrins, mediate numerous developmental processes, particularly in the nervous system. Based on their structures and sequence relationships, ephrins are divided into the ephrin-A (EFNA) class, which are anchored to the membrane by a glycosylphosphatidylinositol linkage, and the ephrin-B (EFNB) class, which are transmembrane proteins. The Eph family of receptors are divided into 2 groups based on the similarity of their extracellular domain sequences and their affinities for binding ephrin-A and ephrin-B ligands. Ephrin receptors make up the largest subgroup of the receptor tyrosine kinase (RTK) family. The protein encoded by this gene is a receptor for ephrin-B family members. [provided by RefSeq] | - | + | + |
| NOTCH1 (4851) | notch 1: This gene encodes a member of the Notch family. Members of this Type 1 transmembrane protein family share structural characteristics including an extracellular domain consisting of multiple epidermal growth factor-like (EGF) repeats, and an intracellular domain consisting of multiple, different domain types. Notch family members play a role in a variety of developmental processes by controlling cell fate decisions. The Notch signaling network is an evolutionarily conserved intercellular signaling pathway which regulates interactions between physically adjacent cells. In Drosophilia, notch interaction with its cell-bound ligands (delta, serrate) establishes an intercellular signaling pathway that plays a key role in development. Homologues of the notch-ligands have also been identified in human, but precise interactions between these ligands and the human notch homologues remain to be determined. This protein is cleaved in the trans-Golgi network, and presented on the cell surface as a heterodimer. This protein functions as a receptor for membrane bound ligands, and may play multiple roles during development. [provided by RefSeq] | - | + | + |
| NOTCH3 (4854) | notch 3: This gene encodes the third discovered human homologue of the Drosophilia melanogaster type I membrane protein notch. In Drosophilia, notch interaction with its cell-bound ligands (delta, serrate) establishes an intercellular signalling pathway that plays a key role in neural development. Homologues of the notch-ligands have also been identified in human, but precise interactions between these ligands and the human notch homologues remains to be determined. Mutations in NOTCH3 have been identified as the underlying cause of cerebral autosomal dominant arteriopathy with subcortical infarcts and leukoencephalopathy (CADASIL). [provided by RefSeq] | - | + | + |
| NR2E1 (7101) | nuclear receptor subfamily 2, group E, member 1: No summary found | - | + | + |
| SIM1  (6492) | single-minded homolog 1 (Drosophila): SIM1 and SIM2 genes are Drosophila single-minded (sim) gene homologs. SIM1 transcript was detected only in fetal kidney out of various adult and fetal tissues tested. Since the sim gene plays an important role in Drosophila development and has peak levels of expression during the period of neurogenesis,it was proposed that the human SIM gene is a candidate for involvement in certain dysmorphic features (particularly the facial and skull characteristics), abnormalities of brain development, and/or mental retardation of Down syndrome. [provided by RefSeq] | - | + | + |
| SLC1A2 (6506) | solute carrier family 1 (glial high affinity glutamate transporter), member 2: This gene encodes a member of a family of solute transporter proteins. The membrane-bound protein is the principal transporter that clears the excitatory neurotransmitter glutamate from the extracellular space at synapses in the central nervous system. Glutamate clearance is necessary for proper synaptic activation and to prevent neuronal damage from excessive activation of glutamate receptors. Mutations in and decreased expression of this protein are associated with amyotrophic lateral sclerosis. Alternatively spliced transcript variants of this gene have been identified. [provided by RefSeq] | - | + | + |
| CNTNAP2 (26047) | contactin associated protein-like 2: This gene encodes a member of the neurexin family which functions in the vertebrate nervous system as cell adhesion molecules and receptors. This protein, like other neurexin proteins, contains epidermal growth factor repeats and laminin G domains. In addition, it includes an F5/8 type C domain, discoidin/neuropilin- and fibrinogen-like domains, thrombospondin N-terminal-like domains and a putative PDZ binding site. This protein is localized at the juxtaparanodes of myelinated axons, and mediates interactions between neurons and glia during nervous system development and is also involved in localization of potassium channels within differentiating axons. This gene encompasses almost 1.5% of chromosome 7 and is one of the largest genes in the human genome. It is directly bound and regulated by forkhead box protein P2 (FOXP2), a transcription factor related to speech and language development. This gene has been implicated in multiple neurodevelopmental disorders, including Gilles de la Tourette syndrome, schizophrenia, epilepsy, autism, ADHD and mental retardation. | - | + | - |
| DAB1  (1600) | disabled homolog 1 (Drosophila): The laminar organization of multiple neuronal types in the cerebral cortex is required for normal cognitive function. In mice, the disabled-1 gene plays a central role in brain development, directing the migration of cortical neurons past previously formed neurons to reach their proper layer. This gene is similar to disabled-1, and the protein encoded by this gene is thought to be a signal transducer that interacts with protein kinase pathways to regulate neuronal positioning in the developing brain. Alternatively spliced transcript variants of this gene have been reported, but their full length nature has not been determined. [provided by RefSeq] | - | + | - |
| DSCAML1 (57453) | Down syndrome cell adhesion molecule like 1: No summary found | - | + | - |
| EN2  (2020) | engrailed homeobox 2: Homeobox-containing genes are thought to have a role in controlling development. In Drosophila, the &apos;engrailed&apos; (en) gene plays an important role during development in segmentation, where it is required for the formation of posterior compartments. Different mutations in the mouse homologs, En1 and En2, produced different developmental defects that frequently are lethal. The human engrailed homologs 1 and 2 encode homeodomain-containing proteins and have been implicated in the control of pattern formation during development of the central nervous system. [provided by RefSeq] | - | + | - |
| ERBB4 (2066) | v-erb-a erythroblastic leukemia viral oncogene homolog 4 (avian): This gene is a member of the Tyr protein kinase family and the epidermal growth factor receptor subfamily. It encodes a single-pass type I membrane protein with multiple cysteine rich domains, a transmembrane domain, a tyrosine kinase domain, a phosphotidylinositol-3 kinase binding site and a PDZ domain binding motif. The protein binds to and is activated by neuregulins and other factors and induces a variety of cellular responses including mitogenesis and differentiation. Multiple proteolytic events allow for the release of a cytoplasmic fragment and an extracellular fragment. Mutations in this gene have been associated with cancer. Alternatively spliced variants which encode different protein isoforms have been described; however, not all variants have been fully characterized. [provided by RefSeq] | - | + | - |
| FOXB1 (27023) | forkhead box B1: No summary found | - | + | - |
| IGFBP1 (3484) | insulin-like growth factor binding protein 1: This gene is a member of the insulin-like growth factor binding protein (IGFBP) family and encodes a protein with an IGFBP domain and a thyroglobulin type-I domain. The protein binds both insulin-like growth factors (IGFs) I and II and circulates in the plasma. Binding of this protein prolongs the half-life of the IGFs and alters their interaction with cell surface receptors. [provided by RefSeq] | - | + | - |
| MDGA1 (266727) | MAM domain containing glycosylphosphatidylinositol anchor 1: No summary found | - | + | - |
| NOS2  (4843) | nitric oxide synthase 2, inducible: Nitric oxide is a reactive free radical which acts as a biologic mediator in several processes, including neurotransmission and antimicrobial and antitumoral activities. This gene encodes a nitric oxide synthase which is expressed in liver and is inducible by a combination of lipopolysaccharide and certain cytokines. Three related pseudogenes are located within the Smith-Magenis syndrome region on chromosome 17. [provided by RefSeq] | - | + | - |
| RELN  (5649) | reelin: This gene encodes a large secreted extracellular matrix protein thought to control cell-cell interactions critical for cell positioning and neuronal migration during brain development. This protein may be involved in schizophrenia, autism, bipolar disorder, major depression and in migration defects associated with temporal lobe epilepsy. Mutations of this gene are associated with autosomal recessive lissencephaly with cerebellar hypoplasia. Two transcript variants encoding distinct isoforms have been identified for this gene. Other transcript variants have been described but their full length nature has not been determined. [provided by RefSeq] | - | + | - |
| SHROOM2 (357) | shroom family member 2: The protein encoded by this gene shares significant similarities with the apical protein from Xenopus laevis which is implicated in amiloride-sensitive sodium channel activity. This gene is a strong candidate gene for ocular albinism type 1 syndrome. [provided by RefSeq] | - | + | - |
| UNC5C (8633) | unc-5 homolog C (C. elegans): This gene product belongs to the UNC-5 family of netrin receptors. Netrins are secreted proteins that direct axon extension and cell migration during neural development. They are bifunctional proteins that act as attractants for some cell types and as repellents for others, and these opposite actions are thought to be mediated by two classes of receptors. The UNC-5 family of receptors mediate the repellent response to netrin; they are transmembrane proteins containing 2 immunoglobulin (Ig)-like domains and 2 type I thrombospondin motifs in the extracellular region. [provided by RefSeq] | - | + | - |
| WNT3A (89780) | wingless-type MMTV integration site family, member 3A: The WNT gene family consists of structurally related genes which encode secreted signaling proteins. These proteins have been implicated in oncogenesis and in several developmental processes, including regulation of cell fate and patterning during embryogenesis. This gene is a member of the WNT gene family. It encodes a protein which shows 96% amino acid identity to mouse Wnt3A protein, and 84% to human WNT3 protein, another WNT gene product. This gene is clustered with WNT14 gene, another family member, in chromosome 1q42 region. [provided by RefSeq] | - | + | - |
|  | **total number of genes** | **49** | **26** | **12** |

Table S13. Functional summary based on the Eldorado database for all individual genes listed in any of the previous tables.

| **gene symbol (ID)** | **functional summary** |
| --- | --- |
| A2ML1 (144568) | The alpha-macroglobulin (AM) superfamily of proteins contains both complement components and protease inhibitors, including A2M (MIM 103950) and A2ML1. AM proteins display a unique trap mechanism of inhibition, by which the AM inhibitor undergoes a major conformational change upon its cleavage by a protease, thus trapping the protease and blocking it from subsequent substrate binding (Galliano et al., 2006 [PubMed 16298998]).[supplied by OMIM] |
| ABCF1  (23) | The protein encoded by this gene is a member of the superfamily of ATP-binding cassette (ABC) transporters. ABC proteins transport various molecules across extra- and intra-cellular membranes. ABC genes are divided into seven distinct subfamilies (ABC1, MDR/TAP, MRP, ALD, OABP, GCN20, White). This protein is a member of the GCN20 subfamily. Unlike other members of the superfamily, this protein lacks the transmembrane domains which are characteristic of most ABC transporters. This protein may be regulated by tumor necrosis factor-alpha and play a role in enhancement of protein synthesis and the inflammation process. |
| ACSBG2 (81616) | No summary found |
| ADAMTS16 (170690) | This gene encodes a member of the ADAMTS (a disintegrin and metalloproteinase with thrombospondin motifs) protein family. ADAMTS family members share several distinct protein modules, including a propeptide region, a metalloproteinase domain, a disintegrin-like domain, and a thrombospondin type 1 (TS) motif. Individual members of this family differ in the number of C-terminal TS motifs, and some have unique C-terminal domains. The protein encoded by this gene has high sequence similarity to the protein encoded by ADAMTS18, another family member. |
| ADORA2A (135) | This gene encodes a protein which is one of several receptor subtypes for adenosine. The activity of the encoded protein, a G-protein coupled receptor family member, is mediated by G proteins which activate adenylyl cyclase. The encoded protein is abundant in basal ganglia, vasculature and platelets and it is a major target of caffeine. |
| ANKRD60 (140731) | No summary found |
| ANKS1A (23294) | No summary found |
| ARRDC4 (91947) | No summary found |
| ART5 (116969) | The protein encoded by this gene belongs to the ARG-specific ADP-ribosyltransferase family. Proteins in this family regulate the function of target proteins by attaching ADP-ribose to specific amino acid residues in their target proteins. The mouse homolog lacks a glycosylphosphatidylinositol-anchor signal sequence and is predicted to be a secretory enzyme. Transcript variants with different 5&apos; UTRs, but encoding the same protein have been found for this gene. |
| ATAD2B (54454) | No summary found |
| ATF7IP (55729) | ATF7IP is a multifunctional nuclear protein that associates with heterochromatin. It can act as a transcriptional coactivator or corepressor depending upon its binding partners (summary by Liu et al., 2009 [PubMed 19106100]) |
| ATP6AP2 (10159) | This gene encodes a protein that is associated with adenosine triphosphatases (ATPases). Proton-translocating ATPases have fundamental roles in energy conservation, secondary active transport, acidification of intracellular compartments, and cellular pH homeostasis. There are three classes of ATPases- F, P, and V. The vacuolar (V-type) ATPases have a transmembrane proton-conducting sector and an extramembrane catalytic sector. The encoded protein has been found associated with the transmembrane sector of the V-type ATPases. |
| ATP6V0D2 (245972) | No summary found |
| BMPER (168667) | No summary found |
| C13orf38-SOHLH2 (100526761) | This locus represents naturally occurring read-through transcription between the neighboring C13orf38 (chromosome 13 open reading frame 38) and SOHLH2 (spermatogenesis and oogenesis specific basic helix-loop-helix 2) genes. The read-through transcript encodes a fusion protein that shares sequence identity with the products of each individual gene. |
| C7 (730) | C7 is a component of the complement system. It participates in the formation of Membrane Attack Complex (MAC). People with C7 deficiency are prone to bacterial infection. |
| CARTPT (9607) | This gene encodes a secreted protein which is processed by prohormone/proprotein convertases to produce smaller, biologically active peptides. Expression of the transcript for this gene is regulated by certain drugs such as cocaine, and the encoded protein is thought to be involved in the regulation of appetite and stress. Mutations in this gene are associated with susceptibility to obesity. |
| CCDC112 (153733) | No summary found |
| CCDC3 (83643) | No summary found |
| CCDC51 (79714) | No summary found |
| CCDC80 (151887) | No summary found |
| CENPN (55839) | The centromere is a specialized chromatin domain, present throughout the cell cycle, that acts as a platform on which the transient assembly of the kinetochore occurs during mitosis. All active centromeres are characterized by the presence of long arrays of nucleosomes in which CENPA (MIM 117139) replaces histone H3 (see MIM 601128). CENPN is an additional factor required for centromere assembly (Foltz et al., 2006 [PubMed 16622419]).[supplied by OMIM] |
| CGA  (1081) | The four human glycoprotein hormones chorionic gonadotropin (CG), luteinizing hormone (LH), follicle stimulating hormone (FSH), and thyroid stimulating hormone (TSH) are dimers consisting of alpha and beta subunits that are associated noncovalently. The alpha subunits of these hormones are identical, however, their beta chains are unique and confer biological specificity. The protein encoded by this gene is the alpha subunit and belongs to the glycoprotein hormones alpha chain family. |
| CNTN1 (1272) | The protein encoded by this gene is a member of the immunoglobulin superfamily. It is a glycosylphosphatidylinositol (GPI)-anchored neuronal membrane protein that functions as a cell adhesion molecule. It may play a role in the formation of axon connections in the developing nervous system. Two alternatively spliced transcript variants encoding different isoforms have been described for this gene. |
| COL12A1 (1303) | This gene encodes the alpha chain of type XII collagen, a member of the FACIT (fibril-associated collagens with interrupted triple helices) collagen family. Type XII collagen is a homotrimer found in association with type I collagen. |
| COL4A3 (1285) | Type IV collagen, the major structural component of basement membranes, is a multimeric protein composed of 3 alpha subunits. These subunits are encoded by 6 different genes, alpha 1 through alpha 6, each of which can form a triple helix structure with 2 other subunits to form type IV collagen. This gene encodes alpha 3. In the Goodpasture syndrome, autoantibodies bind to the collagen molecules in the basement membranes of alveoli and glomeruli. The epitopes that elicit these autoantibodies are localized largely to the non-collagenous C-terminal domain of the protein. A specific kinase phosphorylates amino acids in this same C-terminal region and the expression of this kinase is upregulated during pathogenesis. This gene is also linked to an autosomal recessive form of Alport syndrome. The mutations contributing to this syndrome are also located within the exons that encode this C-terminal region. Like the other members of the type IV collagen gene family, this gene is organized in a head-to-head conformation with another type IV collagen gene so that each gene pair shares a common promoter. [provided by RefSeq] |
| COL4A4 (1286) | This gene encodes one of the six subunits of type IV collagen, the major structural component of basement membranes. This particular collagen IV subunit, however, is only found in a subset of basement membranes. Like the other members of the type IV collagen gene family, this gene is organized in a head-to-head conformation with another type IV collagen gene so that each gene pair shares a common promoter. Mutations in this gene are associated with type II autosomal recessive Alport syndrome (hereditary glomerulonephropathy) and with familial benign hematuria (thin basement membrane disease). Two transcripts, differing only in their transcription start sites, have been identified for this gene and, as is common for collagen genes, multiple polyadenylation sites are found in the 3&apos; UTR. |
| CP  (1356) | The protein encoded by this gene is a metalloprotein that binds most of the copper in plasma and is involved in the peroxidation of Fe(II)transferrin to Fe(III) transferrin. Mutations in this gene cause aceruloplasminemia, which results in iron accumulation and tissue damage, and is associated with diabetes and neurologic abnormalities. |
| CPA4 (51200) | This gene is a member of the carboxypeptidase A/B subfamily, and it is located in a cluster with three other family members on chromosome 7. Carboxypeptidases are zinc-containing exopeptidases that catalyze the release of carboxy-terminal amino acids, and are synthesized as zymogens that are activated by proteolytic cleavage. This gene could be involved in the histone hyperacetylation pathway. It is imprinted and may be a strong candidate gene for prostate cancer aggressiveness. |
| CREBL2 (1389) | cAMP response element (CRE)-binding protein-like-2 (CREBL2) was identified in a search to find genes in a commonly deleted region on chromosome 12p13 flanked by ETV6 and CDKN1B genes, frequently associated with hematopoietic malignancies, as well as breast, non-small-cell lung and ovarian cancers. CREBL2 shares a 41% identity with CRE-binding protein (CREB) over a 48-base long region which encodes the bZip domain of CREB. The bZip domain consists of about 30 amino acids rich in basic residues involved in DNA binding, followed by a leucine zipper motif involved in protein dimerization. This suggests that CREBL2 encodes a protein with DNA binding capabilities. The occurance of CREBL2 deletion in malignancy suggests that CREBL2 may act as a tumor suppressor gene. |
| CRH  (1392) | Corticotropin-releasing hormone is secreted by the paraventricular nucleus (PVN) of the hypothalamus in response to stress. Marked reduction in this protein has been observed in association with Alzheimer disease and autosomal recessive hypothalamic corticotropin deficiency has multiple and potentially fatal metabolic consequences including hypoglycemia and hepatitis. In addition to production in the hypothalamus, this protein is also synthesized in peripheral tissues, such as T lymphocytes and is highly expressed in the placenta. In the placenta it is a marker that determines the length of gestation and the timing of parturition and delivery. A rapid increase in circulating levels of the hormone occurs at the onset of parturition, suggesting that, in addition to its metabolic functions, this protein may act as a trigger for parturition. |
| CST2  (1470) | The cystatin superfamily encompasses proteins that contain multiple cystatin-like sequences. Some of the members are active cysteine protease inhibitors, while others have lost or perhaps never acquired this inhibitory activity. There are three inhibitory families in the superfamily, including the type 1 cystatins (stefins), type 2 cystatins and the kininogens. The type 2 cystatin proteins are a class of cysteine proteinase inhibitors found in a variety of human fluids and secretions, where they appear to provide protective functions. The cystatin locus on chromosome 20 contains the majority of the type 2 cystatin genes and pseudogenes. This gene is located in the cystatin locus and encodes a secreted thiol protease inhibitor found at high levels in saliva, tears and seminal plasma. |
| CT45A5 (441521) | This gene represents one of a cluster of six similar genes located on the q arm of chromosome X. The genes in this cluster encode members of the cancer/testis (CT) family of antigens, and are distinct from other CT antigens. These antigens are thought to be novel therapeutic targets for human cancers. Alternative splicing results in multiple transcript variants. A related pseudogene has been identified on chromosome 5. |
| CXCL11 (6373) | Chemokines are a group of small (approximately 8 to 14 kD), mostly basic, structurally related molecules that regulate cell trafficking of various types of leukocytes through interactions with a subset of 7-transmembrane, G protein-coupled receptors. Chemokines also play fundamental roles in the development, homeostasis, and function of the immune system, and they have effects on cells of the central nervous system as well as on endothelial cells involved in angiogenesis or angiostasis. Chemokines are divided into 2 major subfamilies, CXC and CC. This gene is a CXC member of the chemokine superfamily. Its encoded protein induces a chemotactic response in activated T-cells and is the dominant ligand for CXC receptor-3. The gene encoding this protein contains 4 exons and at least three polyadenylation signals which might reflect cell-specific regulation of expression. IFN-gamma is a potent inducer of transcription of this gene. |
| CYR61 (3491) | CYR61 is a secreted, cysteine-rich, heparin-binding protein encoded by a growth factor-inducible immediate-early gene. Acting as an extracellular, matrix-associated signaling molecule, CYR61 promotes the adhesion of endothelial cells through interaction with integrin and augments growth factor-induced DNA synthesis in the same cell type.[supplied by OMIM] |
| DAXX  (1616) | This gene encodes a multifunctional protein that resides in multiple locations in the nucleus and in the cytoplasm. It interacts with a wide variety of proteins, such as apoptosis antigen Fas, centromere protein C, and transcription factor erythroblastosi |
| DGCR8 (54487) | This gene encodes a subunit of the microprocessor complex which mediates the biogenesis of microRNAs from the primary microRNA transcript. The encoded protein is a double-stranded RNA binding protein that functions as the non-catalytic subunit of the micr |
| DPP4  (1803) | The protein encoded by this gene is identical to adenosine deaminase complexing protein-2, and to the T-cell activation antigen CD26. It is an intrinsic membrane glycoprotein and a serine exopeptidase that cleaves X-proline dipeptides from the N-terminus of polypeptides. |
| EHMT1 (79813) | The protein encoded by this gene is a histone methyltransferase that is part of the E2F6 complex, which represses transcription. The encoded protein methylates the Lys-9 position of histone H3, which tags it for transcriptional repression. This protein may be involved in the silencing of MYC- and E2F-responsive genes and therefore could play a role in the G0/G1 cell cycle transition. Defects in this gene are a cause of chromosome 9q subtelomeric deletion syndrome (9q-syndrome). Two transcript variants encoding different isoforms have been found for this gene. |
| EIF4EBP3 (8637) | This gene encodes a member of the EIF4EBP family, which consists of proteins that bind to eukaryotic translation initiation factor 4E and regulate its assembly into EIF4F, the multi-subunit translation initiation factor that recognizes the mRNA cap structure. Read-through transcription from the neighboring upstream gene (MASK or ANKHD1) generates a transcript (MASK-BP3) that encodes a protein comprised of the MASK protein sequence for the majority of the protein and a different C-terminus due to an alternate reading frame for the EIF4EBP3 segments. |
| ENPP2 (5168) | The protein encoded by this gene functions as both a phosphodiesterase, which cleaves phosphodiester bonds at the 5&apos; end of oligonucleotides, and a phospholipase, which catalyzes production of lysophosphatidic acid (LPA) in extracellular fluids. LPA evokes growth factor-like responses including stimulation of cell proliferation and chemotaxis. This gene product stimulates the motility of tumor cells and has angiogenic properties, and its expression is upregulated in several kinds of carcinomas. The gene product is secreted and further processed to make the biologically active form. Several alternatively spliced transcript variants encoding different isoforms have been identified. |
| EPHA4 (2043) | This gene belongs to the ephrin receptor subfamily of the protein-tyrosine kinase family. EPH and EPH-related receptors have been implicated in mediating developmental events, particularly in the nervous system. Receptors in the EPH subfamily typically have a single kinase domain and an extracellular region containing a Cys-rich domain and 2 fibronectin type III repeats. The ephrin receptors are divided into 2 groups based on the similarity of their extracellular domain sequences and their affinities for binding ephrin-A and ephrin-B ligands. |
| EVI5  (7813) | No summary found |
| FAM114A2 (10827) | No summary found |
| FN1  (2335) | This gene encodes fibronectin, a glycoprotein present in a soluble dimeric form in plasma, and in a dimeric or multimeric form at the cell surface and in extracellular matrix. Fibronectin is involved in cell adhesion and migration processes including embryogenesis, wound healing, blood coagulation, host defense, and metastasis. The gene has three regions subject to alternative splicing, with the potential to produce 20 different transcript variants. However, the full-length nature of some variants has not been determined. |
| GABRA1 (2554) | This gene encodes a gamma-aminobutyric acid (GABA) receptor. GABA is the major inhibitory neurotransmitter in the mammalian brain where it acts at GABA-A receptors, which are ligand-gated chloride channels. Chloride conductance of these channels can be modulated by agents such as benzodiazepines that bind to the GABA-A receptor. GABA-A receptors are pentameric, consisting of proteins from several subunit classes: alpha, beta, gamma, delta and rho. Mutations in this gene cause juvenile myoclonic epilepsy and childhood absence epilepsy type 4. Multiple transcript variants encoding the same protein have been identified for this gene. |
| GABRA3 (2556) | GABA is the major inhibitory neurotransmitter in the mammalian brain where it acts at GABA-A receptors, which are ligand-gated chloride channels. Chloride conductance of these channels can be modulated by agents such as benzodiazepines that bind to the GABA-A receptor. At least 16 distinct subunits of GABA-A receptors have been identified. |
| GATA4 (2626) | This gene encodes a member of the GATA family of zinc-finger transcription factors. Members of this family recognize the GATA motif which is present in the promoters of many genes. This protein is thought to regulate genes involved in embryogenesis and in myocardial differentiation and function. Mutations in this gene have been associated with cardiac septal defects. |
| GDF15 (9518) | Bone morphogenetic proteins (e.g., BMP9; MIM 605120) are members of the transforming growth factor-beta (see TGFB1; MIM 190180) superfamily and regulate tissue differentiation and maintenance. They are synthesized as precursor molecules that are processed at a dibasic cleavage site to release C-terminal domains containing a characteristic motif of 7 conserved cysteines in the mature protein.[supplied by OMIM] |
| GNPDA1 (10007) | Glucosamine-6-phosphate deaminase (EC 3.5.99.6) is an allosteric enzyme that catalyzes the reversible conversion of D-glucosamine-6-phosphate into D-fructose-6-phosphate and ammonium (Arreola et al., 2003 [PubMed 12965206]).[supplied by OMIM] |
| GORASP2 (26003) | This gene encodes a member of the Golgi reassembly stacking protein family. These proteins may play a role in the stacking of Golgi cisternae and Golgi ribbon formation, as well as Golgi fragmentation during apoptosis or mitosis. The encoded protein also plays a role in the intracellular transport of transforming growth factor alpha and may function as a molecular chaperone. A pseudogene of this gene is located on the short arm of chromosome 2. Alternatively spliced transcript variants encoding multiple isoforms have been observed for this gene. |
| GPR139 (124274) | No summary found |
| GRIK5  (2901) | This gene encodes a protein that belongs to the glutamate-gated ionic channel family. Glutamate functions as the major excitatory neurotransmitter in the central nervous system through activation of ligand-gated ion channels and G protein-coupled membrane receptors. The protein encoded by this gene forms functional heteromeric kainate-preferring ionic channels with the subunits encoded by related gene family members. |
| GRPEL2 (134266) | No summary found |
| GSTCD (79807) | No summary found |
| HAS2  (3037) | Hyaluronan or hyaluronic acid (HA) is a high molecular weight unbranched polysaccharide synthesized by a wide variety of organisms from bacteria to mammals, and is a constituent of the extracellular matrix. It consists of alternating glucuronic acid and N-acetylglucosamine residues that are linked by beta-1-3 and beta-1-4 glycosidic bonds. HA is synthesized by membrane-bound synthase at the inner surface of the plasma membrane, and the chains are extruded through pore-like structures into the extracellular space. It serves a variety of functions, including space filling, lubrication of joints, and provision of a matrix through which cells can migrate. HA is actively produced during wound healing and tissue repair to provide a framework for ingrowth of blood vessels and fibroblasts. Changes in the serum concentration of HA are associated with inflammatory and degenerative arthropathies such as rheumatoid arthritis. In addition, the interaction of HA with the leukocyte receptor CD44 is important in tissue-specific homing by leukocytes, and overexpression of HA receptors has been correlated with tumor metastasis. HAS2 is a member of the newly identified vertebrate gene family encoding putative hyaluronan synthases, and its amino acid sequence shows significant homology to glycosaminoglycan synthetase (DG42) from Xenopus laevis, and human and murine hyaluronan synthase 1. [provided by RefSeq] |
| HENMT1 (113802) | No summary found |
| HORMAD1 (84072) | This gene encodes a HORMA domain-containing protein. HORMA domains are involved in chromatin binding and play a role in cell cycle regulation. The encoded protein may play a role in meiosis, and expression of this gene is a potential marker for cancer. A pseudogene of this gene is located on the long arm of chromosome 6. Alternatively spliced transcript variants encoding multiple isoforms have been observed for this gene. |
| ID1  (3397) | The protein encoded by this gene is a helix-loop-helix (HLH) protein that can form heterodimers with members of the basic HLH family of transcription factors. The encoded protein has no DNA binding activity and therefore can inhibit the DNA binding and transcriptional activation ability of basic HLH proteins with which it interacts. This protein may play a role in cell growth, senescence, and differentiation. Two transcript variants encoding different isoforms have been found for this gene. |
| ID2  (3398) | The protein encoded by this gene belongs to the inhibitor of DNA binding family, members of which are transcriptional regulators that contain a helix-loop-helix (HLH) domain but not a basic domain. Members of the inhibitor of DNA binding family inhibit the functions of basic helix-loop-helix transcription factors in a dominant-negative manner by suppressing their heterodimerization partners through the HLH domains. This protein may play a role in negatively regulating cell differentiation. A pseudogene of this gene is located on chromosome 3. |
| ID3  (3399) | Members of the ID family of helix-loop-helix (HLH) proteins lack a basic DNA-binding domain and inhibit transcription through formation of nonfunctional dimers that are incapable of binding to DNA.[supplied by OMIM] |
| ID4  (3400) | Transcription factors containing a basic helix-loop-helix (bHLH) motif regulate expression of tissue-specific genes in a number of mammalian and insect systems. DNA-binding activity of the bHLH proteins is dependent on formation of homo- and/or heterodimers. Dominant-negative HLH proteins encoded by Id-related genes, such as ID4, also contain the HLH-dimerization domain but lack the DNA-binding basic domain. Consequently, Id proteins inhibit binding to DNA and transcriptional transactivation by heterodimerization with bHLH proteins (Pagliuca et al., 1995 [PubMed 7665172]).[supplied by OMIM] |
| IGF2  (3481) | This gene encodes a member of the insulin family of polypeptide growth factors, which are involved in development and growth. It is an imprinted gene, expressed only from the paternal allele, and epigenetic changes at this locus are associated with Wilms tumour, Beckwith-Wiedemann syndrome, rhabdomyosarcoma, and Silver-Russell syndrome. A read-through INS-IGF2 gene exists, whose 5&apos; region overlaps the INS gene and the 3&apos; region overlaps this gene. Alternatively spliced transcript variants encoding different isoforms have been found for this gene. |
| IKZF2 (22807) | This gene encodes a member of the Ikaros family of zinc-finger proteins. Three members of this protein family (Ikaros, Aiolos and Helios) are hematopoietic-specific transcription factors involved in the regulation of lymphocyte development. This protein forms homo- or hetero-dimers with other Ikaros family members, and is thought to function predominantly in early hematopoietic development. Multiple transcript variants encoding different isoforms have been found for this gene, but the biological validity of some variants has not been determined. |
| INS-IGF2 (723961) | This locus includes two alternatively spliced read-through transcript variants which align to the INS gene in the 5&apos; region and to the IGF2 gene in the 3&apos; region. One transcript is predicted to encode a protein which shares the N-terminus with the INS protein but has a distinct and longer C-terminus, whereas the other transcript is a candidate for nonsense-mediated decay (NMD). The transcripts are imprinted and are paternally expressed in the limb and eye. |
| ITGB8  (3696) | This gene is a member of the integrin beta chain family and encodes a single-pass type I membrane protein with a VWFA domain and four cysteine-rich repeats. This protein noncovalently binds to an alpha subunit to form a heterodimeric integrin complex. In general, integrin complexes mediate cell-cell and cell-extracellular matrix interactions and this complex plays a role in human airway epithelial proliferation. Alternatively spliced variants which encode different protein isoforms have been described; however, not all variants have been fully characterized. |
| KALRN (8997) | Huntington&apos;s disease (HD), a neurodegenerative disorder characterized by loss of striatal neurons, is caused by an expansion of a polyglutamine tract in the HD protein huntingtin. This gene encodes a protein that interacts with the huntingtin-associated protein 1, which is a huntingtin binding protein that may function in vesicle trafficking. Alternatively spliced transcript variants encoding different isoforms have been described. |
| KIAA1539 (80256) | No summary found |
| KLHL36 (79786) | No summary found |
| LAMA4 (3910) | Laminins, a family of extracellular matrix glycoproteins, are the major noncollagenous constituent of basement membranes. They have been implicated in a wide variety of biological processes including cell adhesion, differentiation, migration, signaling, neurite outgrowth and metastasis. Laminins are composed of 3 non identical chains: laminin alpha, beta and gamma (formerly A, B1, and B2, respectively) and they form a cruciform structure consisting of 3 short arms, each formed by a different chain, and a long arm composed of all 3 chains. Each laminin chain is a multidomain protein encoded by a distinct gene. Several isoforms of each chain have been described. Different alpha, beta and gamma chain isomers combine to give rise to different heterotrimeric laminin isoforms which are designated by Arabic numerals in the order of their discovery, i.e. alpha1beta1gamma1 heterotrimer is laminin 1. The biological functions of the different chains and trimer molecules are largely unknown, but some of the chains have been shown to differ with respect to their tissue distribution, presumably reflecting diverse functions in vivo. This gene encodes the alpha chain isoform laminin, alpha 4. The domain structure of alpha 4 is similar to that of alpha 3, both of which resemble truncated versions of alpha 1 and alpha 2, in that approximately 1,200 residues at the N-terminus (domains IV, V and VI) have been lost. Laminin, alpha 4 contains the C-terminal G domain which distinguishes all alpha chains from the beta and gamma chains. The RNA analysis from adult and fetal tissues revealed developmental regulation of expression, however, the exact function of laminin, alpha 4 is not known. Tissue-specific utilization of alternative polyA-signal has been described in literature. Alternative splicing results in multiple transcript variants encoding distinct isoforms. [provided by RefSeq] |
| LAMP2 (3920) | The protein encoded by this gene is a member of a family of membrane glycoproteins. This glycoprotein provides selectins with carbohydrate ligands. It may play a role in tumor cell metastasis. It may also function in the protection, maintenance, and adhesion of the lysosome. Alternative splicing of this gene results in multiple transcript variants encoding distinct proteins. |
| LAPTM5 (7805) | This gene encodes a transmembrane receptor that is associated with lysosomes. The encoded protein, also known as E3 protein, may play a role in hematopoiesis. |
| LMO3 (55885) | No summary found |
| LYZ  (4069) | This gene encodes human lysozyme, whose natural substrate is the bacterial cell wall peptidoglycan (cleaving the beta[1-4]glycosidic linkages between N-acetylmuramic acid and N-acetylglucosamine). Lysozyme is one of the anti-microbial agents found in human milk, and is also present in spleen, lung, kidney, white blood cells, plasma, saliva, and tears. Missense mutations in LYZ have been identified in heritable renal amyloidosis. |
| MBD5 (55777) | This gene encodes a member of the methyl-CpG-binding domain (MBD) family. The MBD consists of about 70 residues and is the minimal region required for a methyl-CpG-binding protein binding specifically to methylated DNA. In addition to the MBD domain, this protein contains a PWWP domain (Pro-Trp-Trp-Pro motif), which consists of 100-150 amino acids and is found in numerous proteins that are involved in cell division, growth and differentiation. Mutations in this gene cause mental retardation autosomal dominant type 1. Haploinsufficiency of this gene is associated with a syndrome involving microcephaly, intellectual disabilities, severe speech impairment, and seizures. Alternatively spliced transcript variants have been found, but their full-length nature is not determined. |
| ME1  (4199) | This gene encodes a cytosolic, NADP-dependent enzyme that generates NADPH for fatty acid biosynthesis. The activity of this enzyme, the reversible oxidative decarboxylation of malate, links the glycolytic and citric acid cycles. The regulation of expression for this gene is complex. Increased expression can result from elevated levels of thyroid hormones or by higher proportions of carbohydrates in the diet. |
| METTL7B (196410) | No summary found |
| MID1  (4281) | The protein encoded by this gene is a member of the tripartite motif (TRIM) family, also known as the 'RING-B box-coiled coil' (RBCC) subgroup of RING finger proteins. The TRIM motif includes three zinc-binding domains, a RING, a B-box type 1 and a B-box type 2, and a coiled-coil region. This protein forms homodimers which associate with microtubules in the cytoplasm. The protein is likely involved in the formation of multiprotein structures acting as anchor points to microtubules. Mutations in this gene have been associated with the X-linked form of Opitz syndrome, which is characterized by midline abnormalities such as cleft lip, laryngeal cleft, heart defects, hypospadias, and agenesis of the corpus callosum. This gene was also the first example of a gene subject to X inactivation in human while escaping it in mouse. Multiple different transcript variants are generated by alternate splicing; however, the full-length nature of some of the variants has not been determined. |
| MMP16 (4325) | Proteins of the matrix metalloproteinase (MMP) family are involved in the breakdown of extracellular matrix in normal physiological processes, such as embryonic development, reproduction, and tissue remodeling, as well as in disease processes, such as arthritis and metastasis. Most MMP&apos;s are secreted as inactive proproteins which are activated when cleaved by extracellular proteinases. The encoded protein activates MMP2 by cleavage. This gene was once referred to as MT-MMP2, but was renamed as MT-MMP3 or MMP16. |
| MMP2  (4313) | Proteins of the matrix metalloproteinase (MMP) family are involved in the breakdown of extracellular matrix in normal physiological processes, such as embryonic development, reproduction, and tissue remodeling, as well as in disease processes, such as arthritis and metastasis. Most MMP&apos;s are secreted as inactive proproteins which are activated when cleaved by extracellular proteinases. This gene encodes an enzyme which degrades type IV collagen, the major structural component of basement membranes. The enzyme plays a role in endometrial menstrual breakdown, regulation of vascularization and the inflammatory response. Mutations in this gene have been associated with Winchester syndrome and Nodulosis-Arthropathy-Osteolysis (NAO) syndrome. Two transcript variants encoding different isoforms have been found for this gene. |
| MUT  (4594) | This gene encodes the mitochondrial enzyme methylmalonyl Coenzyme A mutase. In humans, the product of this gene is a vitamin B12-dependent enzyme which catalyzes the isomerization of methylmalonyl-CoA to succinyl-CoA, while in other species this enzyme may have different functions. Mutations in this gene may lead to various types of methylmalonic aciduria. |
| MYO1D (4642) | No summary found |
| NFIC  (4782) | The protein encoded by this gene belongs to the CTF/NF-I family. These are dimeric DNA-binding proteins, and function as cellular transcription factors and as replication factors for adenovirus DNA replication. Alternatively spliced transcript variants encoding different isoforms have been described for this gene. |
| NOX4 (50507) | This gene encodes a member of the NOX-family of enzymes that functions as the catalytic subunit the NADPH oxidase complex. The encoded protein is localized to non-phagocytic cells where it acts as an oxygen sensor and catalyzes the reduction of molecular oxygen to various reactive oxygen species (ROS). The ROS generated by this protein have been implicated in numerous biological functions including signal transduction, cell differentiation and tumor cell growth. A pseudogene has been identified on the other arm of chromosome 11. Alternative splicing results in multiple transcript variants. |
| NUPL1 (9818) | This gene encodes a member of the nucleoporin family that shares 87% sequence identity with rat nucleoporin p58. The protein is localized to the nuclear rim and is a component of the nuclear pore complex (NPC). All molecules entering or leaving the nucleus either diffuse through or are actively transported by the NPC. Alternate transcriptional splice variants, encoding different isoforms, have been characterized. |
| OBSCN (84033) | The obscurin gene spans more than 150 kb, contains over 80 exons and encodes a protein of approximately 720 kDa. The encoded protein contains 68 Ig domains, 2 fibronectin domains, 1 calcium/calmodulin-binding domain, 1 RhoGEF domain with an associated PH domain, and 2 serine-threonine kinase domains. This protein belongs to the family of giant sacromeric signaling proteins that includes titin and nebulin, and may have a role in the organization of myofibrils during assembly and may mediate interactions between the sarcoplasmic reticulum and myofibrils. Alternatively spliced transcript variants encoding different isoforms have been identified. |
| ODZ1 (10178) | The protein encoded by this gene belongs to the tenascin family and teneurin subfamily. It is expressed in the neurons and may function as a cellular signal transducer. Several alternatively spliced transcript variants encoding different isoforms have been found for this gene. |
| PCBD2 (347732) | No summary found |
| PGK1  (5230) | The protein encoded by this gene is a glycolytic enzyme that catalyzes the conversion of 1,3-diphosphoglycerate to 3-phosphoglycerate. The encoded protein may also act as a cofactor for polymerase alpha. This gene lies on the X-chromosome, while a related pseudogene also has been found on the X-chromosome and another on chromosome 19. |
| PIGK  (10026) | This gene encodes a member of the cysteine protease family C13 that is involved in glycosylphosphatidylinositol (GPI)-anchor biosynthesis. The GPI-anchor is a glycolipid found on many blood cells and serves to anchor proteins to the cell surface. This protein is a member of the multisubunit enzyme, GPI transamidase and is thought to be its enzymatic component. GPI transamidase mediates GPI anchoring in the endoplasmic reticulum, by catalyzing the transfer of fully assembled GPI units to proteins. |
| PLEK  (5341) | No summary found |
| PLK2  (10769) | Serum-inducible kinase is a member of the &apos;polo&apos; family of serine/threonine protein kinases that have a role in normal cell division.[supplied by OMIM] |
| PLRG1 (5356) | This gene encodes a core component of the cell division cycle 5-like (CDC5L) complex. The CDC5L complex is part of the spliceosome and is required for pre-mRNA splicing. The encoded protein plays a critical role in alternative splice site selection. Alternatively spliced transcript variants encoding multiple isoforms have been observed for this gene. |
| PMPCB (9512) | This gene is a member of the peptidase M16 family and encodes a protein with a zinc-binding motif. This protein is located in the mitochondrial matrix and catalyzes the cleavage of the leader peptides of precursor proteins newly imported into the mitochondria, though it only functions as part of a heterodimeric complex. |
| PNMA3 (29944) | This gene is a member of the paraneoplastic antigen MA (PNMA) gene family, whose protein products share homology with retroviral Gag proteins. They are highly expressed in the brain and also in a range of tumors associated with serious neurological phenotypes. PMID:16407312 reports the presence of a functional -1 ribosomal frameshift signal (consisting of a heptanucleotide shift motif followed 3' by a pseudoknot structure) in this gene, however, the frame-shifted product has not been characterized. |
| PODXL (5420) | This gene encodes a member of the sialomucin protein family. The encoded protein was originally identified as an important component of glomerular podocytes. Podocytes are highly differentiated epithelial cells with interdigitating foot processes covering the outer aspect of the glomerular basement membrane. Other biological activities of the encoded protein include: binding in a membrane protein complex with Na+/H+ exchanger regulatory factor to intracellular cytoskeletal elements, playing a role in hematopoetic cell differentiation, and being expressed in vascular endothelium cells and binding to L-selectin. |
| POPDC3 (64208) | This gene encodes a member of the POP family of proteins containing three putative transmembrane domains. This gene is expressed in cardiac and skeletal muscle and may play an important role in these tissues during development. Alternatively spliced transcript variants have been found. |
| POT1 (25913) | This gene is a member of the telombin family and encodes a nuclear protein involved in telomere maintenance. Specifically, this protein functions as a member of a multi-protein complex that binds to the TTAGGG repeats of telomeres, regulating telomere length and protecting chromosome ends from illegitimate recombination, catastrophic chromosome instability, and abnormal chromosome segregation. Increased transcriptional expression of this gene is associated with stomach carcinogenesis and its progression. Alternatively spliced transcript variants have been described. |
| PREX1 (57580) | The protein encoded by this gene acts as a guanine nucleotide exchange factor for the RHO family of small GTP-binding proteins (RACs). It has been shown to bind to and activate RAC1 by exchanging bound GDP for free GTP. The encoded protein, which is found mainly in the cytoplasm, is activated by phosphatidylinositol-3,4,5-trisphosphate and the beta-gamma subunits of heterotrimeric G proteins. |
| PSMD10 (5716) | This gene encodes a subunit of the PA700/19S complex, which is the regulatory component of the 26S proteasome. The 26S proteosome complex is required for ubiquitin-dependent protein degradation. This protein is a non-ATPase subunit that may be involved in protein-protein interactions. Aberrant expression of this gene may paly a role in tumorigenesis. Two transcripts encoding different isoforms have been described. Pseudogenes have been identified on chromosomes 3 and 20. |
| PTDSS1 (9791) | Phosphatidylserine (PS) accounts for 5 to 10% of cell membrane phospholipids. In addition to its role as a structural component, PS is involved in cell signaling, blood coagulation, and apoptosis. PS is synthesized by a calcium-dependent base-exchange reaction catalyzed by PS synthases (EC 2.7.8.8), like PTDSS1, that exchange L-serine for the polar head group of phosphatidylcholine (PC) or phosphatidylethanolamine (PE) (Sturbois-Balcerzak et al., 2001 [PubMed 11084049]).[supplied by OMIM] |
| PTGFR (5737) | The protein encoded by this gene is member of the G-protein coupled receptor family. This protein is a receptor for prostaglandin F2-alpha (PGF2-alpha), which is known to be a potent luteolytic agent, and may also be involved in modulating intraocular pressure and smooth muscle contraction in uterus. Knockout studies in mice suggest that the interaction of PGF2-alpha with this receptor may initiate parturition in ovarian luteal cells and thus induce luteolysis. Two transcript variants encoding different isoforms have been found for this gene. |
| RAPGEF5 (9771) | Members of the RAS (see HRAS; MIM 190020) subfamily of GTPases function in signal transduction as GTP/GDP-regulated switches that cycle between inactive GDP- and active GTP-bound states. Guanine nucleotide exchange factors (GEFs), such as RAPGEF5, serve as RAS activators by promoting acquisition of GTP to maintain the active GTP-bound state and are the key link between cell surface receptors and RAS activation (Rebhun et al., 2000 [PubMed 10934204]).[supplied by OMIM] |
| RNF14 (9604) | The protein encoded by this gene contains a RING zinc finger, a motif known to be involved in protein-protein interactions. This protein interacts with androgen receptor (AR) and may function as a coactivator that induces AR target gene expression in prostate. A dominant negative mutant of this gene has been demonstrated to inhibit the AR-mediated growth of prostate cancer. This protein also interacts with class III ubiquitin-conjugating enzymes (E2s) and may act as a ubiquitin-ligase (E3) in the ubiquitination of certain nuclear proteins. Six alternatively spliced transcript variants encoding two distinct isoforms have been reported. |
| S100A13 (6284) | The protein encoded by this gene is a member of the S100 family of proteins containing 2 EF-hand calcium-binding motifs. S100 proteins are localized in the cytoplasm and/or nucleus of a wide range of cells, and involved in the regulation of a number of cellular processes such as cell cycle progression and differentiation. S100 genes include at least 13 members which are located as a cluster on chromosome 1q21. This protein is widely expressed in various types of tissues with a high expression level in thyroid gland. In smooth muscle cells, this protein co-expresses with other family members in the nucleus and in stress fibers, suggesting diverse functions in signal transduction. Multiple alternatively spliced transcript variants encoding the same protein have been found for this gene. |
| SAGE1 (55511) | This gene belongs to a class of genes that are activated in tumors. These genes are expressed in tumors of different histologic types but not in normal tissues, except for spermatogenic cells and, for some, placenta. The proteins encoded by these genes appear to be strictly tumor specific, and hence may be excellent sources of antigens for cancer immunotherapy. This gene is expressed in sarcomas. |
| SAMD11 (148398) | No summary found |
| SCARNA5 (677775) | No summary found |
| SCG2  (7857) | The protein encoded by this gene is a member of the chromogranin/secretogranin family of neuroendocrine secretory proteins. Studies in rodents suggest that the full-length protein, secretogranin II, is involved in the packaging or sorting of peptide hormones and neuropeptides into secretory vesicles. The full-length protein is cleaved to produce the active peptide secretoneurin, which exerts chemotaxic effects on specific cell types, and EM66, whose function is unknown. |
| SEC31A (22872) | The protein encoded by this gene is similar to yeast Sec31 protein. Yeast Sec31 protein is known to be a component of the COPII protein complex which is responsible for vesicle budding from endoplasmic reticulum (ER). This protein was found to colocalize with SEC13, one of the other components of COPII , in the subcellular structures corresponding to the vesicle transport function. An immunodepletion experiment confirmed that this protein is required for ER-Golgi transport. Alternative splicing results in multiple transcript variants encoding different isoforms. |
| SEMA3C (10512) | No summary found |
| SEPP1 (6414) | This gene encodes a selenoprotein containing multiple selenocysteine (Sec) residues, which are encoded by the UGA codon that normally signals translation termination. The 3&apos; UTR of selenoprotein genes have a common stem-loop structure, the sec insertion sequence (SECIS), which is necessary for the recognition of UGA as a Sec codon rather than as a stop signal. This selenoprotein is an extracellular glycoprotein, and is unusual in that it contains 10 Sec residues per polypeptide. It is a heparin-binding protein that appears to be associated with endothelial cells, and has been implicated to function as an antioxidant in the extracellular space. Several transcript variants, encoding either the same or different isoform, have been found for this gene. |
| SERPINB9 (5272) | PI9 belongs to the large superfamily of serine proteinase inhibitors (serpins), which bind to and inactivate serine proteinases. These interactions are involved in many cellular processes, including coagulation, fibrinolysis, complement fixation, matrix remodeling, and apoptosis (Sprecher et al., 1995 [PubMed 8530382]).[supplied by OMIM] |
| SGK1  (6446) | This gene encodes a serine/threonine protein kinase that plays an important role in cellular stress response. This kinase activates certain potassium, sodium, and chloride channels, suggesting an involvement in the regulation of processes such as cell survival, neuronal excitability, and renal sodium excretion. High levels of expression of this gene may contribute to conditions such as hypertension and diabetic nephropathy. Several alternatively spliced transcript variants encoding different isoforms have been noted for this gene. |
| SHANK2 (22941) | This gene encodes a protein that is a member of the Shank family of synaptic proteins that may function as molecular scaffolds in the postsynaptic density (PSD). Shank proteins contain multiple domains for protein-protein interaction, including ankyrin repeats, an SH3 domain, a PSD-95/Dlg/ZO-1 domain, a sterile alpha motif domain, and a proline-rich region. This particular family member contains a PDZ domain, a consensus sequence for cortactin SH3 domain-binding peptides and a sterile alpha motif. The alternative splicing demonstrated in Shank genes has been suggested as a mechanism for regulating the molecular structure of Shank and the spectrum of Shank-interacting proteins in the PSDs of adult and developing brain. Two alternative splice variants, encoding distinct isoforms, are reported. Additional splice variants exist but their full-length nature has not been determined. |
| SHISA2 (387914) | No summary found |
| SKIL  (6498) | No summary found |
| SLC13A4 (26266) | No summary found |
| SMAD7 (4092) | The protein encoded by this gene is a nuclear protein that binds the E3 ubiquitin ligase SMURF2. Upon binding, this complex translocates to the cytoplasm, where it interacts with TGF-beta receptor type-1 (TGFBR1), leading to the degradation of both the encoded protein and TGFBR1. Expression of this gene is induced by TGFBR1. Variations in this gene are a cause of susceptibility to colorectal cancer type 3 (CRCS3). Several transcript variants encoding different isoforms have been found for this gene. |
| SNHG12 (85028) | No summary found |
| SNORA14A (677801) | Small nucleolar RNAs (snoRNAs) are 60-150 nt long non-coding RNAs, and include two groups: C/D box snoRNAs and H/ACA box snoRNAs. The C/D box snoRNAs are guides for the 2&apos;-O-ribose methylation of rRNAs or snRNAs. The H/ACA box snoRNAs are guides for the isomerization of uridine residues into pseudouridine. This gene belongs to the group of the H/ACA box snoRNAs, and functions in 18S rRNA pseudouridylation at position U966. The gene duplication generates a 91% identical copy on chromosome 1. |
| SNORA14B (677802) | Small nucleolar RNAs (snoRNAs) are 60-150 nt long non-coding RNAs, and include two groups: C/D box snoRNAs and H/ACA box snoRNAs. The C/D box snoRNAs are guides for the 2&apos;-O-ribose methylation of rRNAs or snRNAs. The H/ACA box snoRNAs are guides for the isomerization of uridine residues into pseudouridine. This gene belongs to the group of the H/ACA box snoRNAs, and functions in 18S rRNA pseudouridylation at position U966. The gene duplication generates a 91% identical copy on chromosome 7. |
| SNORA16A (692073) | No summary found |
| SNORA28 (677811) | No summary found |
| SNORA30 (677813) | No summary found |
| SNORA33 (594839) | No summary found |
| SNORA46 (677827) | No summary found |
| SNORA75 (654321) | No summary found |
| SNORD94 (692225) | No summary found |
| SNX12 (29934) | This gene encodes a member of the sorting nexin family. Members of this family contain a phox (PX) domain, which is a phosphoinositide binding domain, and are involved in intracellular trafficking. This protein does not contain a coiled coil region, like some family members. A similar protein in mouse may be involved in regulating the neurite outgrowth. Alternate splicing results in multiple transcript variants. |
| SOX4  (6659) | This intronless gene encodes a member of the SOX (SRY-related HMG-box) family of transcription factors involved in the regulation of embryonic development and in the determination of the cell fate. The encoded protein may act as a transcriptional regulator after forming a protein complex with other proteins, such as syndecan binding protein (syntenin). The protein may function in the apoptosis pathway leading to cell death as well as to tumorigenesis and may mediate downstream effects of parathyroid hormone (PTH) and PTH-related protein (PTHrP) in bone development. The solution structure has been resolved for the HMG-box of a similar mouse protein. |
| SPARC (6678) | Secreted protein acidic and rich in cysteine/osteonectin/BM40, or SPARC, is a matrix-associated protein that elicits changes in cell shape, inhibits cell-cycle progression, and influences the synthesis of extracellular matrix (ECM) (Bradshaw et al., 2003 [PubMed 12721366]).[supplied by OMIM] |
| SRBD1 (55133) | No summary found |
| STC1  (6781) | This gene encodes a secreted, homodimeric glycoprotein that is expressed in a wide variety of tissues and may have autocrine or paracrine functions. The gene contains a 5' UTR rich in CAG trinucleotide repeats. The encoded protein contains 11 conserved cysteine residues and is phosphorylated by protein kinase C exclusively on its serine residues. The protein may play a role in the regulation of renal and intestinal calcium and phosphate transport, cell metabolism, or cellular calcium/phosphate homeostasis. Overexpression of human stanniocalcin 1 in mice produces high serum phosphate levels, dwarfism, and increased metabolic rate. This gene has altered expression in hepatocellular, ovarian, and breast cancers. |
| STEAP2 (261729) | This gene is a member of the STEAP family and encodes a multi-pass membrane protein that localizes to the Golgi complex, the plasma membrane, and the vesicular tubular structures in the cytosol. A highly similar protein in mouse has both ferrireductase and cupric reductase activity, and stimulates the cellular uptake of both iron and copper in vitro. Increased transcriptional expression of the human gene is associated with prostate cancer progression. Alternate transcriptional splice variants, encoding different isoforms, have been characterized. |
| TJP3  (27134) | TJP3 is a member of the family of membrane-associated guanylate kinase-like proteins (MAGUK) that associate with intracellular junctions (Itoh et al., 1999 [PubMed 10601346]).[supplied by OMIM] |
| TMX1 (81542) | TXNDC1 is a thioredoxin (TXN; see MIM 187700)-related protein with disulfide reductase activity (Matsuo et al., 2001 [PubMed 11152479]).[supplied by OMIM] |
| TNFRSF10D (8793) | The protein encoded by this gene is a member of the TNF-receptor superfamily. This receptor contains an extracellular TRAIL-binding domain, a transmembrane domain, and a truncated cytoplamic death domain. This receptor does not induce apoptosis, and has been shown to play an inhibitory role in TRAIL-induced cell apoptosis. |
| TNIK (23043) | Germinal center kinases (GCKs), such as TNIK, are characterized by an N-terminal kinase domain and a C-terminal GCK domain that serves a regulatory function (Fu et al., 1999 [PubMed 10521462]).[supplied by OMIM] |
| TOB1 (10140) | This gene encodes a member of the tob/btg1 family of anti-proliferative proteins that have the potential to regulate cell growth. When exogenously expressed, this protein supresses cell growth in tissue culture. The protein undergoes phophorylation by a serine/threonine kinase, 90 kDa ribosomal S6 kinase. Interactions of this protein with the v-erb-b2 erythroblastic leukemia viral oncogene homolog 2 gene product p185 interferes with growth suppression. This protein inhibits T cell proliferation and transcription of cytokines and cyclins. The protein interacts with both mothers against decapentaplegic Drosophila homolog 2 and 4 to enhance their DNA binding activity. This interaction inhibits interleukin 2 transcription in T cells. |
| TRMT1L (81627) | This gene encodes a protein that has some similarity to N2,N2-dimethylguanosine tRNA methyltransferase from other organisms. Studies of the mouse ortholog have shown that this protein plays a role in motor coordination and exploratory behavior, and it may also be involved in modulating postnatal neuronal functions. Alternatively spliced transcripts have been identified for this gene. |
| TSTD2 (158427) | No summary found |
| TUBE1 (51175) | This gene encodes a member of the tubulin superfamily. This protein localizes to the centriolar sub-distal appendages that are associated with the older of the two centrioles after centrosome duplication. This protein plays a central role in organization of the microtubules during centriole duplication. A pseudogene of this gene is found on chromosome 5. |
| USPL1 (10208) | No summary found |
| VAV1  (7409) | The protein encoded by this proto-oncogene is a member of the Dbl family of guanine nucleotide exchange factors (GEF) for the Rho family of GTP binding proteins. The protein is important in hematopoiesis, playing a role in T-cell and B-cell development and activation. This particular GEF has been identified as the specific binding partner of Nef proteins from HIV-1. Coexpression and binding of these partners initiates profound morphological changes, cytoskeletal rearrangements and the JNK/SAPK signaling cascade, leading to increased levels of viral transcription and replication. |
| WDR31 (114987) | This gene encodes a member of the WD repeat protein family. WD repeats are minimally conserved regions of approximately 40 amino acids typically bracketed by gly-his and trp-asp (GH-WD), which may facilitate formation of heterotrimeric or multiprotein complexes. Members of this family are involved in a variety of cellular processes, including cell cycle progression, signal transduction, apoptosis, and gene regulation. Multiple alternatively spliced transcript variants encoding distinct isoforms have been found for this gene but the biological validity of some variants has not been determined. |
| YKT6 (10652) | This gene product is one of the SNARE recognition molecules implicated in vesicular transport between secretory compartments. It is a membrane associated, isoprenylated protein that functions at the endoplasmic reticulum-Golgi transport step. This protein |
| ZBTB16 (7704) | This gene is a member of the Krueppel C2H2-type zinc-finger protein family and encodes a zinc finger transcription factor that contains nine Kruppel-type zinc finger domains at the carboxyl terminus. This protein is located in the nucleus, is involved in cell cycle progression, and interacts with a histone deacetylase. Specific instances of aberrant gene rearrangement at this locus have been associated with acute promyelocytic leukemia (APL). Alternate transcriptional splice variants have been characterized. |
| ZBTB33 (10009) | This gene encodes a transcriptional regulator with bimodal DNA-binding specificity, which binds to methylated CGCG and also to the non-methylated consensus KAISO-binding site TCCTGCNA. The protein contains an N-terminal POZ/BTB domain and 3 C-terminal zinc finger motifs. It recruits the N-CoR repressor complex to promote histone deacetylation and the formation of repressive chromatin structures in target gene promoters. It may contribute to the repression of target genes of the Wnt signaling pathway, and may also activate transcription of a subset of target genes by the recruitment of catenin delta-2 (CTNND2). Its interaction with catenin delta-1 (CTNND1) inhibits binding to both methylated and non-methylated DNA. It also interacts directly with the nuclear import receptor Importin-&amp;#945;2 (also known as karyopherin alpha2 or RAG cohort 1), which may mediate nuclear import of this protein. Alternatively spliced transcript variants encoding the same protein have been identified. |
| ZNF80 (7634) | No summary found |

**Supporting references**

1. . [Melnyk RA](http://www.ncbi.nlm.nih.gov/pubmed?term="Melnyk RA"%5BAuthor%5D), [Tam J](http://www.ncbi.nlm.nih.gov/pubmed?term="Tam J"%5BAuthor%5D), [Boie Y](http://www.ncbi.nlm.nih.gov/pubmed?term="Boie Y"%5BAuthor%5D), [Kennedy BP](http://www.ncbi.nlm.nih.gov/pubmed?term="Kennedy BP"%5BAuthor%5D), [Percival MD](http://www.ncbi.nlm.nih.gov/pubmed?term="Percival MD"%5BAuthor%5D) (2009) Renin and prorenin activate pathways implicated in organ damage in human mesangial cells independent of angiotensin II production. Am J Nephrol 30: 232-243. [↑](#endnote-ref-2)
2. . [He M](http://www.ncbi.nlm.nih.gov/pubmed?term="He M"%5BAuthor%5D), [Zhang L](http://www.ncbi.nlm.nih.gov/pubmed?term="Zhang L"%5BAuthor%5D), [Shao Y](http://www.ncbi.nlm.nih.gov/pubmed?term="Shao Y"%5BAuthor%5D), [Wang X](http://www.ncbi.nlm.nih.gov/pubmed?term="Wang X"%5BAuthor%5D), [Huang Y](http://www.ncbi.nlm.nih.gov/pubmed?term="Huang Y"%5BAuthor%5D), [Yao T](http://www.ncbi.nlm.nih.gov/pubmed?term="Yao T"%5BAuthor%5D), [Lu L](http://www.ncbi.nlm.nih.gov/pubmed?term="Lu L"%5BAuthor%5D). (2009) Inhibition of renin/prorenin receptor attenuated mesangial cell proliferation and reduced associated fibrotic factor release. [Eur J Pharmacol](http://www.ncbi.nlm.nih.gov/pubmed/?term=He et al. Inhibition of renin/prorenin ) 606:155-161. [↑](#endnote-ref-3)
3. . Clavreul N, Sansilvestri-Morel P, Magard D, Verbeuren TJ, Rupin A. (2011) (Pro)renin promotes fibrosis gene expression in HEK cells through a Nox4-dependent mechanism. Am J Physiol Renal Physiol 300: F1310-1318. [↑](#endnote-ref-4)
4. . [Saris JJ](http://www.ncbi.nlm.nih.gov/pubmed?term="Saris JJ"%5BAuthor%5D), ['t Hoen PA](http://www.ncbi.nlm.nih.gov/pubmed?term="'t Hoen PA"%5BAuthor%5D), [Garrelds IM](http://www.ncbi.nlm.nih.gov/pubmed?term="Garrelds IM"%5BAuthor%5D), [Dekkers DH](http://www.ncbi.nlm.nih.gov/pubmed?term="Dekkers DH"%5BAuthor%5D), [den Dunnen JT](http://www.ncbi.nlm.nih.gov/pubmed?term="den Dunnen JT"%5BAuthor%5D), et al. (2006) Prorenin induces intracellular signaling in cardiomyocytes independently of angiotensin II. [Hypertension](http://www.ncbi.nlm.nih.gov/pubmed/16940215) 48: 564-571. [↑](#endnote-ref-5)
5. . Riediger F, Quack I, Qadri F, Hartleben B, Park JK, et al. (2011) Prorenin receptor is essential for podocyte autophagy and survival. J Am Soc Nephrol 22: 2193-2202. [↑](#endnote-ref-6)
6. . Oshima Y, Kinouchi K, Ichihara A, Sakoda M, Kurauchi-Mito A, et al. (2011) Prorenin receptor is essential for normal podocyte structure and function. J Am Soc Nephrol 22: 2203-2212. [↑](#endnote-ref-7)
7. . Wang N, Frank GD, Ding R, Tan Z, Rachakonda A, et al. (2012) Promyelocytic leukemia zinc finger protein activates GATA4 transcription and mediates cardiac hypertrophic signaling from angiotensin II receptor 2. PLoS ONE 7: e35632. [↑](#endnote-ref-8)
8. . [Bernardo MV](http://www.ncbi.nlm.nih.gov/pubmed?term="Bernardo MV"%5BAuthor%5D), [Yelo E](http://www.ncbi.nlm.nih.gov/pubmed?term="Yelo E"%5BAuthor%5D), [Gimeno L](http://www.ncbi.nlm.nih.gov/pubmed?term="Gimeno L"%5BAuthor%5D), [Campillo JA](http://www.ncbi.nlm.nih.gov/pubmed?term="Campillo JA"%5BAuthor%5D), [Parrado A](http://www.ncbi.nlm.nih.gov/pubmed?term="Parrado A"%5BAuthor%5D) (2007) Identification of apoptosis-related PLZF target genes. [Biochem Biophys Res Commun](http://www.ncbi.nlm.nih.gov/pubmed?term=Bernardo 2007 plzf) 359: 317-322. [↑](#endnote-ref-9)
9. . [Doulatov S](http://www.ncbi.nlm.nih.gov/pubmed?term="Doulatov S"%5BAuthor%5D), [Notta F](http://www.ncbi.nlm.nih.gov/pubmed?term="Notta F"%5BAuthor%5D), [Rice KL](http://www.ncbi.nlm.nih.gov/pubmed?term="Rice KL"%5BAuthor%5D), [Howell L](http://www.ncbi.nlm.nih.gov/pubmed?term="Howell L"%5BAuthor%5D), [Zelent A](http://www.ncbi.nlm.nih.gov/pubmed?term="Zelent A"%5BAuthor%5D), et al. (2009) PLZF is a regulator of homeostatic and cytokine-induced myeloid development. [Genes Dev.](http://www.ncbi.nlm.nih.gov/pubmed/19723763) 23: 2076-2087. [↑](#endnote-ref-10)
